# Gut–Brain–Microbiome Axis in the Regulation of Cancer Immune Escape and Immunotherapy in Tumors

**DOI:** 10.34133/research.0885

**Published:** 2025-09-11

**Authors:** Wangting Xu, Qifan Xu, Peng Luo, Xudong Qu, Dandan Guo, Zhao Xie, Na Hang, Minjie Kuang, Enguo Chen, Ling Wang, Zhiping Yan, Songhua Cai, Wenjun Mao, Bufu Tang

**Affiliations:** ^1^ Department of Pulmonary and Critical Care Medicine, Affiliated Sir Run Run Shaw Hospital, Zhejiang University School of Medicine, Hangzhou, China.; ^2^ Dalian Medical University, Dalian, China.; ^3^ Department of Oncology Zhujiang Hospital, Southern Medical University, Guangzhou, Guangdong, China.; ^4^ Department of Interventional Radiology, Zhongshan Hospital, Shanghai Institute of Medical Imaging, National Clinical Research Center of Interventional Medicine, Fudan University, Shanghai, China.; ^5^ University of Edinburgh Institute, Zhejiang University School of Medicine, Ningbo, Zhejiang, China.; ^6^ Department of Thoracic Surgery, National Cancer Center/National Clinical Research Center for Cancer/Cancer Hospital & Shenzhen Hospital, Chinese Academy of Medical Sciences and Peking Union Medical College, Shenzhen, Guangdong, China.; ^7^ Department of Thoracic Surgery, The Affiliated Wuxi People’s Hospital of Nanjing Medical University, Wuxi People’s Hospital, Wuxi Medical Center, Nanjing Medical University, Wuxi, China.

## Abstract

Accumulating evidence demonstrates crosstalk involving the intestine and the brain, with the gut microbiota serving as a critical mediator of this interaction. The gut microbiota, along with its derived metabolites and bioactive compounds, modulates the immune microenvironment of brain tumors via the gut–brain axis, thereby influencing tumor initiation, progression, invasion, and metastasis. This review systematically summarizes the pathophysiological mechanisms underlying gut–brain axis modulation of brain tumors and examines emerging therapeutic approaches, including advances in immunotherapy and targeted therapy, that hold promise for future brain tumor treatment strategies.

Central nervous system (CNS) tumors exhibit exceptionally high lethality, characterized by extensive tumor heterogeneity, therapeutic resistance, and blood–brain barrier (BBB) restrictions, which collectively compromise the efficacy of conventional treatments, resulting in poor prognosis and low survival rates. Emerging evidence highlights that the microbiota–gut–brain axis plays a crucial role in modulating the immune microenvironment of CNS tumors and promoting tumor progression, thereby presenting a promising therapeutic target for immunological and targeted interventions. Accordingly, it is imperative to innovate in therapeutic approaches through more profound insight into the microbiota–gut–brain axis to address these challenges in CNS tumor management.

## Neuro-Oncological Immunology

### Nervous system neoplasms

#### Classification, incidence, and epidemiological characteristics of nervous system tumors

Primary intracranial malignancies have a high mortality rate and constitute a major health burden [1]. In Table 1, we present the classification and incidence of CNS tumors. Gliomas, the predominant category of brain tumors, originate in glial cells and manifest across all age groups, demonstrating higher prevalence in adults and males [2]. Adult gliomas are classified into molecular subtypes with varying outcomes, with isocitrate dehydrogenase (IDH) wild-type glioblastomas (GBMs) being the most lethal [3]. Roughly 90% of GBMs exhibit IDH wild-type status, and they are classified as World Health Organization (WHO) grade IV tumors, predominantly affecting older individuals and characterized by aggressive progression and poor prognosis [4]. Primary central nervous system lymphoma (PCNSL), histologically classified as large B cell lymphoma, typically develops in the brain, spinal cord, leptomeninges, or eyes [5], characterized by high invasiveness, poor prognosis, and frequent recurrence. The most common molecular subtypes of ependymoma include posterior fossa group B ependymoma (EPN-PFB), posterior fossa subependymoma (PF-SE), and supratentorial ependymoma with ZFTA fusion (EPN-ZFTA) [6].

**Table 1.** Classification and incidence of central nervous system tumors

Tumor type	Tumor subtype	Incidence	Five-year survival rate	Current treatment	Reference
Adult diffuse infiltrating low-grade gliomas	IDH mutated and 1p/19q co-deleted oligodendrogliomas	30%	36%	Combination therapy of procarbazine, lomustine, and vincristine with radiotherapy	[1,3,4,7]
	IDH mutated astrocytomas				
	IDH wild-type glioblastomas			Temozolomide combined with radiotherapy	
Primary central nervous system lymphomas	B cell lymphoma	7%	30%–40%	High-dose methotrexate induction followed by thiotepa-conditioned transplantation, whole-brain radiotherapy, or single-agent maintenance consolidation	[1,5]
Malignant ependymoma	EPN-PFB	3%	64.5%	Maximal safe resection whenever possible; for WHO grade 3 tumors or subtotally resected grade 2 tumors, local radiotherapy is administered	[1,4,6]
	PF-SE		67.4%		
	EPN-ZFTA		60.3%		
Meningiomas	WHO grade I (meningothelial, fibrous variants and 7 other variants)	2%	86.1%	Resection or stereotactic radiosurgery	[1,4,65,225]
	WHO grade II (3 variants)		75.9%–93.6%		
	WHO grade III (3 variants)		66%		

Glioblastomas, particularly the IDH wild-type subtype, account for 49% of cases, while diffuse infiltrating low-grade gliomas make up 30%, including IDH-mutant oligodendrogliomas and astrocytomas. Other tumor types include primary central nervous system lymphomas (7%), malignant ependymomas (3%), and meningiomas (2%), with further subtypes based on molecular and anatomical characteristics.

#### Treatment modalities for nervous system neoplasms

Standard treatment for nervous system tumors involves surgery, alkylating chemotherapy, and radiotherapy, with specific regimens like temozolomide (TMZ) or PCV (procarbazine, lomustine, and vincristine) plus radiotherapy improving survival in GBM and 1p/19q co-deleted oligodendrogliomas [1]. However, even with postoperative radiotherapy and chemotherapy, the improvement in survival for glioma patients remains limited to approximately 3 months [7]. The standard treatment paradigm for PCNSL comprises induction chemotherapy with high-dose methotrexate, followed by consolidation with large-dose cytarabine and radiotherapy for whole brain or autologous stem cell transplantation, with or without single-agent maintenance therapy. Unfortunately, approximately 15% to 25% of patients exhibit primary resistance to standard chemotherapy regimens, and among those who initially respond to treatment, 25% to 50% eventually experience relapse [5]. For malignant ependymomas, maximal safe surgical resection should be pursued when feasible; however, focal radiotherapy is indicated for grade III tumors or unresectable grade II lesions. Current treatment strategies remain insufficient to substantially reduce the risk of recurrence, which most often occurs within 24 months of diagnosis [8]. The management of malignant meningiomas typically involves surgical resection or stereotactic radiosurgery. The recurrence rate of grade 2 and grade 3 meningiomas remains notably high, ranging from 50% to 90% [1]. This underscores the fact that current treatment options for various types of brain tumors are still limited. Due to the BBB, which effectively blocks nearly all large-molecule drugs and approximately 98% of small-molecule compounds from entering brain tissue, the therapeutic efficacy of existing drugs is substantially constrained, contributing to persistently high tumor recurrence rates [7]. Therefore, the development of novel treatment strategies has become an urgent priority.

### Nervous system immune microenvironment

#### The CNS microenvironment: Structure and function

The CNS microenvironment comprises specialized cell types, including neurons, endothelial cells, pericytes, immune cells, and glial cells [9]. The BBB, established by the neurovascular unit (NVU) [10], serves as a protective interface between the bloodstream and neural tissue, shielding the brain from external threats but also significantly restricting drug delivery [11]. Among glial cells, oligodendrocytes are responsible for maintaining myelin sheaths and supporting neuronal function. While astrocytes and microglia orchestrate coordinated responses to brain injury, astrocytes enhance BBB integrity through hypertrophy and scar formation, whereas microglia become activated to clear cellular debris [12].

#### CNS immune responses: Cells and cytokines

The CNS immune system involves diverse immune and neural cell types, including macrophages, lymphocytes, and astrocytes, alongside cytokines and immune checkpoint molecules [13]. Border-associated macrophages (BAMs) [14], located at CNS interfaces like the leptomeninges and choroid plexus [15], play key roles in neuroimmune responses by producing inflammatory cytokines [e.g., interleukin-1β (IL-1β) and tumor necrosis factor-α (TNF-α)] and antigen-presenting molecules, increasing BBB permeability, and recruiting granulocytes [16]. They also modulate systemic anti-inflammatory pathways via cyclooxygenase-2 (COX2) and prostaglandin E2 (PGE2).

CNS-resident T lymphocyte subsets vary in location and function, influencing either neuroprotection or damage depending on cytokine secretion, with interferon-γ (IFN-γ) and IL-4 imbalances disrupting CNS homeostasis [13]. Regulatory T cells (Tregs) suppress the pyroptosis of microglia through the Toll-like receptor 4 (TLR4)/MyD88/nuclear factor κB (NF-κB) signaling cascade in demyelination induced by LPC, consequently ameliorating myelin loss and enhancing cognitive function [17].

Although B cells are few in number in the CNS, they exert significant immunological effects by producing antibodies, secreting inflammatory cytokines and their inhibitors, and influencing CNS pathophysiology through both peripheral and local immune responses [18].

Neutrophils are largely excluded from the healthy CNS due to the BBB, but under pathological conditions, they can infiltrate and contribute to BBB breakdown [19].

Mast cells are key CNS-resident immune sensors that detect microbes and cell damage through pattern recognition receptors. Following pathogenic stimulation, mast cells undergo degranulation, releasing abundant inflammatory mediators that propagate immune signals and activate adjacent glial cells, thereby initiating neuroinflammatory cascades [20]. While this response may facilitate tissue repair, persistent damage can trigger sustained pro-inflammatory mediator release, resulting in chronic inflammation that ultimately culminates in neuronal dysfunction and death [20].

Natural killer (NK) cells, though limited in number, reside mainly in the CNS parenchyma and perform immune surveillance, with both neuroprotective and neurotoxic roles in CNS diseases [21].

γδ T cells are innate-like lymphocytes that bridge innate and adaptive immunity in the CNS [22], influencing disease pathogenesis through pattern recognition, rapid inflammatory mediator release, and cytokine-driven immunoregulation [23].

Due to limited immune cell access, cytokines play a central role in CNS immunity. Their dysregulation can trigger inflammation and tissue damage, disrupting CNS homeostasis. This review highlights key cytokines involved in CNS immune regulation. Specifically, IL-34, synthesized by CNS-resident cells, orchestrates microglial growth under physiological conditions and promotes BBB integrity through the up-regulation of tight junction proteins [24]. IL-33, steadily expressed in the CNS, protects against tissue damage and promotes repair [24]. TNF-α, mainly from CD8^+^ T cells, regulates neurogenesis, myelination, and BBB integrity, but excessive levels can cause excitotoxicity and neuroinflammation [25]. The function of immune cells in CNS is shown in Table 2.

**Table 2.** Immune cells in CNS and their functions

Immune cell	Roles in CNS	Reference
BAMs (macrophages and monocytes)	Mediate neuronal damage; mediate systemic anti-inflammatory response	[13,15,16]
T cells	Secrete cytokines with either protective or pathogenic effects on the central nervous system (CNS); monitor pathogens; maintain cognitive and social behavior; inhibit oligodendrocyte pyroptosis to reduce myelin loss and improve cognition	[13,17]
B cells	Synthesize antibodies; secrete cytokines that promote and inhibit inflammation; promote CNS pathology	[18]
Neutrophils	Release neutrophil extracellular traps to damage the BBB and neurons	[19]
Mast cells	Release granules to release mast cell mediators, activate other neuroglial cells, and initiate inflammation; release pro-inflammatory mediators to amplify inflammation	[20]
NK cells	Participate in immune surveillance; play a dual role in neuroprotection and neurotoxicity	[21]
γδT cells	Recognize antigens; promptly generate inflammatory mediators; secrete cytokines to influence antibody differentiation	[23]

Table 2 outlines the roles of various immune cells in the CNS. BAMs, T cells, B cells, and neutrophils contribute to neuroinflammation, neuronal damage, and BBB disruption, while mast cells amplify immune responses by releasing inflammatory mediators. NK cells and γδ T cells play dual roles in immune surveillance, neuroprotection, and neurotoxicity, influencing CNS pathology through cytokine secretion and antigen recognition.

#### Tumor immune microenvironment

The CNS tumor microenvironment (TME) includes various immune cells, with T lymphocytes being the dominant tumor-infiltrating lymphocytes (TILs) [26]. However, cytotoxic T cells often become dysfunctional due to GBM-induced apoptosis, immunosuppressive cytokines [e.g., transforming growth factor-β (TGF-β) and IL-10] [27], and immune checkpoint up-regulation (e.g., PD-1, LAG-3, and TIGIT) [26]. T cell infiltration is further limited by retention in bone marrow [28], and Treg accumulation is driven by indoleamine 2,3-dioxygenase-1-expressing (IDO1^+^) dendritic cells (DCs) and CCR4–CCL22 signaling [29]. This immunosuppressive environment hinders antitumor responses. Additionally, γδ T cells in the CNS TME display NK-like features and recognize antigens independently of major histocompatibility complex (MHC) [30]. γδ T lymphocytes possess the capacity to detect cellular stress signals and diverse antigenic molecules, including phosphoantigens and lipid moieties, thereby facilitating immunosurveillance [31]. In a seminal study, Choi and colleagues [32] demonstrated using an orthotopic mouse model that intratumoral administration of allogeneic γδ T lymphocytes elicits anti-GBM responses through DNAX accessory molecule-1 (DNAM-1)-dependent mechanisms. Notably, hypoxic conditions attenuate the antitumor functionality of γδ T lymphocytes, whereas mitigation of cerebral oxygen consumption or hypoxia-inducible factor-1α (HIF-1α) inhibition in these cells can restore their cytotoxic capacity [33].

Tumor-associated macrophages (TAMs), mainly exhibiting an immunosuppressive M2 phenotype, are abundant in the CNS TME and promote tumor growth, angiogenesis, and metastasis [34]. Their polarization is driven by various factors, including osteopontin from mesenchymal-like endothelial cells [35], glioblastoma stem cell (GSC)-derived signals (e.g., exosomal miR-200c-3p and CXCL8) [36], lactate, and CCL2/CD84–SHP2 signaling [37]. M2 TAMs enhance glioma invasion via TLR2-induced matrix metalloproteinase-14 (MMP-14)/MMP-2 expression and support GSC proliferation [38], epithelial–mesenchymal transition (EMT), and therapy resistance. Their infiltration correlates with tumor grade and recurrence, with cluster of differentiation 47 (CD47) signaling aiding immune evasion [39]. Macrophage-derived CD47 provides an inhibitory “don’t eat me” message, facilitating meningioma immune evasion and disease progression [40]. TAMs also impair T cell function (e.g., via TBC1D1) [41] and cooperate with GSCs through PTN–PTPRZ1 signaling [42]. Beyond classical M2 types, novel TAM subsets like lipid-laden macrophages (LLMs) [43] and hypoxia-adapted Mo-TAMs have been identified, contributing to tumor metabolism and vascular dysfunction, and are associated with poor prognosis [44].

**Fig. 1.**
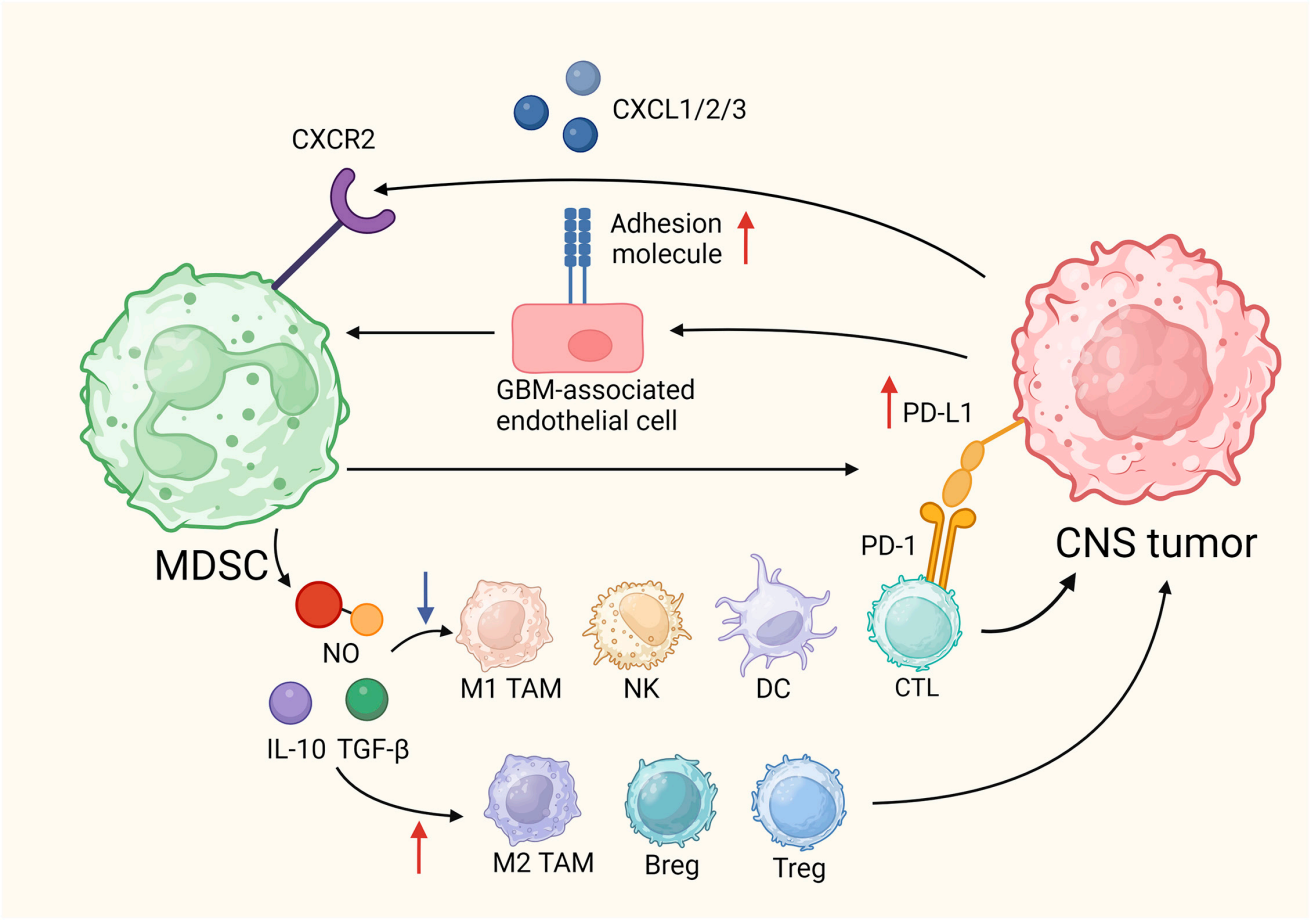
The function of MDSCs within the immunosuppressive milieu of CNS tumors.

Myeloid-derived suppressor cells (MDSCs) play a key immunosuppressive role in the CNS TME, shown in Fig. 1. In GBM, tumor-derived CXCL1/2/3 and CXCR2 promote polymorphonuclear myeloid-derived suppressor cell (PMN-MDSC) mobilization and expansion [45]. After chemoradiotherapy, increased endothelial adhesion molecules enhance MDSC recruitment [46]. MDSCs suppress cytotoxic immune cells via nitric oxide (NO), IL-10, TGF-β, and PD-L1 while promoting Tregs, regulatory B cells (Bregs), and M2 TAMs [46]. Their infiltration correlates with higher tumor grade and worse prognosis [26].

MDSC accumulation occurs within the GBM microenvironment through the interaction of tumor-derived chemokines (CXCL1/2/3) with their receptor CXCR2. Chemoradiotherapy enhances this recruitment by up-regulating adhesion molecules on GBM-associated endothelial cells. Within the tumor microenvironment, MDSCs exert immunosuppressive effects by releasing nitric oxide (NO), IL-10, and TGF-β. The mediators inhibit cytotoxic effector cells, including CD8^+^ T lymphocytes, NK cells, M1 TAMs, and DCs, while promoting immunosuppressive populations including Tregs, Bregs, and M2-polarized TAMs (M2 TAMs). Additionally, MDSCs up-regulate PD-L1, leading to T cell exhaustion and immune evasion, thereby driving tumor progression. Red arrows indicate up-regulation, while blue arrows represent down-regulation.

NK cells play a key role in the CNS TME by targeting GBM cells with low MHC expression through cytotoxicity and apoptosis induction [28]. While activated NK cells are generally associated with better GBM prognosis [26], they may paradoxically promote glioma progression via IFN-γ [47]. In GBM, tumor cells suppress NK cell function by down-regulating activating receptors and up-regulating immunosuppressive molecules like CD73 and MHC-I [28].

CNS neutrophils originate from nearby bone marrow and enter tumors as tumor-associated neutrophils (TANs) [48], with N1 TANs suppressing tumors and N2 TANs promoting progression [28]. In early GBM, TANs are mostly antitumor N1 cells but shift to pro-tumor N2 cells as the disease advances, driven by hypoxia and acrolein [49]. Neutrophils also contribute to GBM growth via neutrophil extracellular trap (NET) formation through HMGB1/RAGE/IL-8 signaling [50], while LC3-associated phagocytosis can induce tumor cell ferroptosis and necroptosis [51].

DCs play key roles in the CNS TME, with mature DC1s promoting antitumor immunity. However, in high-grade gliomas (HGGs), factors like vascular endothelial growth factor (VEGF) and IL-10 impair DC function and promote immunosuppressive regulatory DCs (rDCs) that activate Tregs and inhibit T cells [26]. GBM stem-like cells can further drive malignant DC transformation via ZNF148/PTX3 signaling [52], while tumor-derived exosomes induce ferroptosis in DCs through modulation of the NRF2/glutathione peroxidase-4 (GPX4) pathway, contributing to tumor progression [53].

### Predictive models for neurological tumors

Advances in AI, including machine learning and radiogenomics, have enabled predictive models that enhance CNS tumor diagnosis, treatment planning, and outcome prediction, offering new tools for clinical management and future therapies. Emerging evidence supports predictive models as effective CNS tumor diagnostic tools. The DEPLOY model was not only trained and cross-validated using internal datasets but also validated across 3 independent external clinical cohorts, including the Digital Brain Tumor Atlas (DBTA), the Children’s Brain Tumor Network (CBTN), and a National Cancer Institute (NCI) prospective cohort. These validations encompassed data from over 2,100 patients and demonstrated exceptionally high overall accuracy and strong potential for clinical application. However, several limitations remain before the model can be widely implemented in clinical settings. These include its current applicability being restricted to only 10 types of CNS tumors, reduced classification accuracy for rare subtypes with small sample sizes, and heavy reliance on methylation-matched data during the training process [54]. Joo et al. [55] developed a magnetic resonance imaging (MRI)-based radiogenomic model using edema volume, radiomic features, and demographics to accurately predict meningeal tumor brain invasion, improving diagnostic precision beyond standard imaging methods.

A range of advanced predictive models is offering new avenues for precision therapy in glioma. Molecular omics-based models—including a cuproptosis activity scoring system, ferroptosis-related gene signatures, classifications defined by immune cell infiltration states, and circulating tumor DNA (ctDNA)-based models—can effectively identify potential therapeutic targets, evaluate tumor aggressiveness and survival risk, and predict recurrence location and treatment response. For example, Chen et al. [56] constructed a cuproptosis activity scoring system to assess glioma aggressiveness and prognosis; Han et al. [57] developed a prognostic model based on ferroptosis-related genes and identified SLC1A5 as a key biomarker; Nam et al. [58] subtyped ependymoma using immune cell infiltration features and found that subtype A was associated with superior survival; and Heger et al. [59] developed a ctDNA-based predictive model for risk assessment in CNS lymphoma.

In addition, advances in artificial intelligence (AI) and radiomics show promising potential in brain tumor management. For instance, Herrgott et al. [60] leveraged AI with DNA methylation data to predict treatment outcomes in meningioma; Zhou et al. [61] proposed a fusion model that achieved precise tumor segmentation and recurrence prediction; and Fan et al. [62] introduced RadSurv, a radiomics-derived survival biomarker that enables risk stratification in glioma and is associated with M2 macrophage infiltration as well as predicted responsiveness to immunotherapy.

## Host Immune Responses within the Gut Microbiota–CNS Tumor Axis

**Fig. 2.**
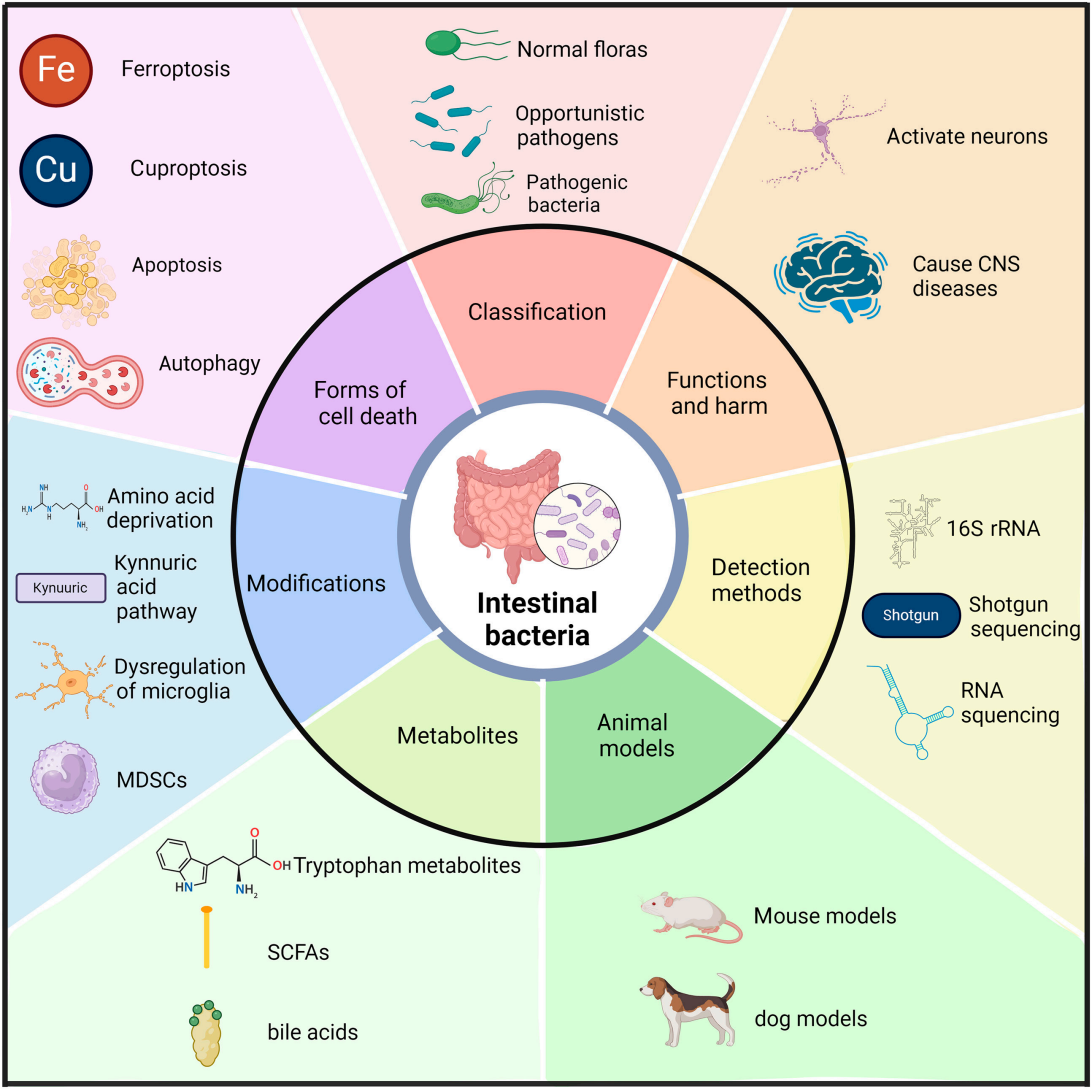
Overview of intestinal bacteria and their associated functions, detection methods, metabolites, modifications, and roles in cell death.

Intestinal bacteria can be categorized based on pathogenicity into normal flora, opportunistic pathogens, and pathogenic bacteria. These bacteria contribute to various functions and harm, such as directly activating neurons or causing CNS diseases. Detection methods for intestinal bacteria incorporate shotgun metagenomic sequencing, 16*S* ribosomal RNA (rRNA) gene sequencing, and RNA sequencing. Animal models, such as mouse and dog models, are commonly used to study intestinal microbiota. Key metabolites produced by intestinal bacteria include tryptophan-derived metabolites, short-chain fatty acids (SCFAs), and bile acids. Modifications of intestinal bacteria involve mechanisms such as amino acid deprivation (e.g., tryptophan and arginine), the kynurenic acid pathway, dysregulation of microglia, and the involvement of MDSCs. Furthermore, intestinal bacteria are implicated in multiple cell death modalities, including ferroptosis, cuproptosis, autophagy, and apoptosis. The key points outlined above are comprehensively summarized in Fig. 2 for an overall overview.

### Fundamentals and characteristics of the intestinal microbiota

#### Taxonomic hierarchy and functional diversity of the intestinal microbiota: Current classification systems

The human gastrointestinal tract hosts a complex ecosystem of over 100 trillion microorganisms, collectively known as the gut microbiota. The predominant phyla, Bacteroidetes and Firmicutes, constitute approximately 90% of the gut microbiome, representing the core microbial community. At the taxonomic level of genus, Bacteroidetes encompasses *Bacteroides* and *Prevotella*, whereas Firmicutes comprises *Clostridium*, *Enterococcus*, *Lactobacillus*, and *Faecalibacterium* [63,64].

From a functional and pathogenicity perspective, the gut microbiota can be categorized into 3 distinct groups: commensals, opportunistic pathogens, and pathogens. Commensals comprise microorganisms that maintain a mutually beneficial relationship with the host, conferring advantages through biological antagonism, nutritional support, immune modulation, and metabolic regulation. Representative commensal genera include *Bifidobacterium*, *Lactobacillus*, and *Enterococcus*. Opportunistic pathogens represent microorganisms that typically maintain commensalism but may induce pathological conditions under specific circumstances, exemplified by *Escherichia coli*, *Salmonella*, and *Shigella*. Pathogens encompass microorganisms that directly compromise host health, as illustrated by *Helicobacter pylori* [65]. Notably, universally beneficial bacteria are seldom documented in clinical settings, as perturbations in bacterial populations can disrupt microbial homeostasis, leading to dysbiosis and associated pathologies. The ultimate impact of specific bacterial populations on host health is determined by the complex interplay of host–microbiota and microbiota–microbiota interactions [65].

#### Physiological functions and pathological effects of the intestinal microbiota

Commensal bacteria orchestrate the ontogeny of the local intestinal immunity, regulate intestinal sensory and motor functions, facilitate the degradation of complex dietary compounds, enhance nutrient absorption, and synthesize essential vitamins. Select commensal species, particularly *Lactobacillus* and *Bifidobacterium*, employ competitive exclusion mechanisms to prevent pathogenic colonization. These microorganisms, in conjunction with their metabolites, particularly SCFAs, maintain epithelial cell adhesion and strengthen the intestinal barrier function [66]. Recent studies have demonstrated that early gut microbiota can modulate intestinal immune responses through microbially derived acetate lipids, significantly reducing susceptibility to colitis and regulating ferroptosis-related pathways [67]. Conversely, dysbiosis can compromise intestinal barrier integrity, resulting in the formation of epithelial discontinuities. This barrier dysfunction facilitates microbial translocation beyond the intestinal lumen, triggering excessive immune cell activation and profound pro-inflammatory cytokine release, potentially inducing inflammation in both intestinal and extra-intestinal tissues [68].

The intestinal microbiome, through bidirectional communication via the gut–brain axis, exerts profound influences on neurogenesis and neurodevelopment. The intestinal microbiome and its associated metabolites modulate BBB function through up-regulation of tight junction proteins, consequently regulating BBB permeability. These microbial factors affect the evolution and performance of CNS-resident immune cells and mediate direct neuronal activation [66,69]. Studies consistently demonstrate that intestinal microbiome dysregulation represents a significant susceptibility factor in various neurological diseases, encompassing autism spectrum disorder, Alzheimer’s disease, Parkinson’s disease, multiple sclerosis, stroke, anxiety disorders, and schizophrenia [70,71]. Furthermore, recent studies have demonstrated that maternal gut microbiota not only affects maternal neurodevelopment but also significantly modulates offspring gut microbiota colonization through both direct and indirect pathways, thereby influencing the neurodevelopment of offspring [72]. Notably, studies have demonstrated that transcranial magnetic stimulation (TMS) can ameliorate cognitive impairment induced by a high-fat diet in rats by modulating gut microbiota dysbiosis. This finding further elucidates the critical role of the gut–brain axis in the regulation of neurological functions. Although repetitive transcranial magnetic stimulation (rTMS) has demonstrated benefits for cognitive function and intestinal microbiota homeostasis in metabolic disease models, its role in CNS tumors remains unclear. The brain–gut axis may exert dual, environment-dependent effects. On the one hand, it may suppress the initiation and progression of brain tumors by mitigating neuroinflammation. On the other hand, it may promote tumor progression by modulating the tumor immune microenvironment in a manner that favors tumor development [73].

#### Advanced methodologies for gut microbiota analysis

The gut microbiome represents a complex microbial ecosystem that harbors a genetic repertoire exceeding that of the human host by approximately 100-fold, fundamentally influencing host metabolism, immunity, and health status. Given that the majority of gut microorganisms remain recalcitrant to traditional cultivation methods, next-generation sequencing (NGS) technologies have emerged as essential tools in gut microbiome research [74]. The primary NGS methodologies encompass 16*S* rRNA gene sequencing, shotgun metagenomics sequencing, as well as RNA sequencing, each possessing distinct advantages and limitations.

16*S* rRNA sequencing represents a widely adopted methodology for bacterial taxonomic identification. This approach encompasses polymerase chain reaction (PCR) amplification of the bacterial 16*S* rRNA region, followed by high-throughput sequencing of the amplified products. Shotgun metagenomics sequencing initiates with DNA extraction from environmental samples, followed by random fragmentation and subsequent ligation of molecular barcodes and adapters to facilitate multiplexed sequencing. The generated sequence reads undergo quality control processing and subsequent alignment against reference databases for taxonomic classification and functional annotation. RNA sequencing employs methodology similar to shotgun metagenomics; however, it incorporates an additional step wherein RNA fragments undergo reverse transcription to complementary DNA (cDNA) prior to sequencing library preparation.

16*S* rRNA sequencing offers several advantages, including reduced risk of host contamination, minimized false positives, and enhanced cost-efficiency. Limitations of this approach include restricted detection to bacteria, reduced taxonomic resolution at the species level, inability to resolve strain-level variations, absence of direct functional profiling capabilities, increased susceptibility to PCR bias, and complex bioinformatics requirements. In contrast, shotgun metagenomics and RNA sequencing provide comprehensive detection capabilities extending beyond bacteria to include fungi, parasites, and viruses, with RNA sequencing specifically enabling RNA virus detection. These methods analyze the complete microbial genomic content, enabling comprehensive functional characterization—shotgun metagenomics reveals the genetic potential through analysis of encoded genes, while RNA sequencing elucidates active gene expression profiles. Both approaches offer enhanced taxonomic resolution to species and strain levels, facilitate precise functional analysis, and enable discovery of novel microbial taxa. These methods are limited by increased computational requirements and higher operational costs [75]. We provided a comprehensive summary of the major sequencing methods for gut microbiota, covering their types, workflows, advantages, and limitations, as illustrated in Table 3.

**Table 3.** The types, steps, advantages, and disadvantages of sequencing methods for gut microbiota

Types of sequencing method	Sequencing step	Advantage	Disadvantage	Reference
16*S* rRNA sequencing	Amplify bacterial 16*S* ribosomal RNA by PCR and then sequence the resulting products	Low risk of host contamination; low risk of false positives; efficient and cost-effective	Can only detect bacteria; limited resolution for species-level differentiation; limitations to directly obtain functional profiles; higher risk of bias; difficult to perform bioinformatics analysis	[75,76]
Metagenomic shotgun sequencing	Extract the given sample, randomly fragment the DNA, attach barcodes and adapters to both ends of each fragment, clean and sequence the results, and then align them with a reference database	Can detect bacteria, fungi, parasites, and viruses; provided a random subset of microbiome-encoded genes; broader classification coverage; more accurate functional analysis; has the potential to detect unknown microbial species and strains	Higher cost and bioinformatics burden	
RNA sequencing	After fragmentation, RNA fragments are reverse transcribed into cDNA via PCR, and then processed through a DNA sequencing pipeline	Can detect bacteria, fungi, parasites, viruses, and RNA viruses; broader classification coverage; more accurate functional analysis; has the potential to detect unknown microbial species and strains	Higher cost and bioinformatics burden	

Table 3 summarizes the key sequencing methods for gut microbiota, highlighting their processes, advantages, and limitations. While 16*S* rRNA sequencing is cost-effective and minimizes host contamination, it lacks species-level resolution and functional profiling, whereas metagenomic and RNA sequencing provide comprehensive taxonomic and functional insights but require higher costs and computational resources.

#### Experimental animal models for investigating gut microbiota

The intestinal microbiome and host organisms maintain a complex mutualistic relationship, mediated through bidirectional signaling pathways of the gut–brain axis, which orchestrates the ontogeny and function of immune, endocrine, and nervous systems. These physiological interactions are fundamentally linked to health and disease states, as microbiota composition significantly influences behavioral patterns, and dysbiosis potentially precipitates neurological disorders. Experimental animal models have emerged as essential platforms for investigating gut–brain axis functionality, yielding valuable insights into host–microbiota interactions [76].

Two principal experimental paradigms—germ-free (GF) mice and antibiotic-mediated gut dysbiosis models—facilitate the investigation of causal relationships between the gut microbiome and brain function [77].

GF mice, characterized by the absence of endogenous microbiota, exhibit increased populations of immature microglia across multiple brain regions, a phenomenon corroborated by studies utilizing antibiotic-treated mice. Administration of bacterially derived SCFAs to GF mice demonstrates restoration of microglial morphology and function, indicating that microglial-mediated immune programming is dependent on microbial metabolites. Supplementation of GF mice with a defined consortium of 4 *Bifidobacterium* species reveals bacterial regulation of microglial development and activation via transcriptional mechanisms. Collectively, these findings demonstrate the gut microbiota’s essential role in microglial development, maturation, and functional activation. As microglia constitute the primary innate immune cells within the brain, these findings suggest that gut microbiota modulation of microglial function may contribute to the pathogenesis of various CNS disorders. Additionally, GF mice demonstrate compromised BBB integrity, characterized by reduced tight junction protein expression, indicating that gut microbiota potentially influences CNS pathology through modulation of BBB permeability [76].

To elucidate the impact of chronic antibiotic treatment on gut microbiota composition and subsequent glioma progression, mice received 2 parenteral antibiotics. Following a 2-week treatment period, syngeneic GL261 glioma cells were stereotactically implanted into the brain. Tumor volume was quantified after 3 weeks of continuous antibiotic administration. Results demonstrated that antibiotic treatment induced significant alterations in the gut microbiome, leading to modified gut–immune–brain signaling. These alterations resulted in decreased cytotoxic NK cell populations and dysregulated microglial inflammatory and homeostatic protein expression, establishing a CNS microenvironment that promotes tumor tolerance and facilitates glioma progression [78]. In a parallel syngeneic glioma model, antibiotic administration profoundly affected both gut and brain metabolomic signatures. The TME exhibited a pro-angiogenic phenotype characterized by enhanced microglial and glioma cell activation. Enhanced glioma stem cell properties and their transdifferentiation capacity into endothelial progenitor cells augmented angiogenic processes. Elevated glycine levels specifically modulated the brain microenvironment, promoting glioma growth and progression [79].

Canine models represent valuable platforms for gut microbiota research, owing to their greater taxonomic and functional similarity to the human microbiome compared with murine models. In a systematic evaluation of ketogenic diets (KDs), Allenspach et al. [80] administered sequential dietary interventions to 8 healthy adult beagle dogs: a baseline diet followed by 2 distinct KDs (KD1 and KD2, featured by low-carbohydrate, high-fat, and protein-adequate compositions) for 2-week periods. Analysis revealed significant alterations in the canine fecal microbiome, characterized by enrichment of specific taxa (e.g., *Fusobacteria* and *Bifidobacteria*) and depletion of others (e.g., *Lactobacilli*). Further analyses demonstrated that diet-induced dysbiosis correlated with altered microbial metabolic functions, particularly in cholesterol/steroid metabolism and bile acid biosynthesis pathways, indicating potential therapeutic efficacy against glycolipid-dependent cancer stem cells. Moreover, KDs significantly modulated metabolite profiles, reducing valine and methionine levels while elevating serotonin and decreasing kynurenine (Kyn) concentrations, collectively contributing to antitumor effects.

In addition to traditional animal models, 3D bioprinting technology is being actively explored for studying host–microbiota interactions. Recent advances have led to the development of a 3-dimensional (3D) bioprinted intestinal model that offers several advantages over conventional intestinal organoids. This model enables precise control of tissue geometry to enhance reproducibility, better mimics the hypoxic conditions of the human intestinal tract, and allows coculture of intestinal microbiota and epithelial cells to closely replicate their interactions. By employing sacrificial layer printing techniques, a continuous open intestinal lumen has been created, along with complex structures such as villi, crypts, and tight junctions. Looking ahead, we envision that integrating BBB organoids with this 3D bioprinted intestinal model will establish a novel platform to investigate the mechanisms underlying the “gut microbiota–brain–brain tumor” axis. This system holds the potential to more accurately simulate the human microphysiological environment, elucidate how gut microbiota may influence the initiation and progression of brain tumors via the brain–gut axis, and provide a high-throughput, controllable in vitro model for drug screening and mechanistic studies. Ultimately, this approach could accelerate the development of innovative therapeutic strategies targeting the gut microbiome for brain tumor intervention [81].

### Metabolic products of the gut microbiota: Role in neuro-oncology

Gut microbiota-derived metabolites function as critical mediators between the gut microbiome and neurological tumor pathogenesis. These metabolites exert their effects through TME remodeling and regulation of key signaling cascades in both neoplastic and immune cells [82]. Principal among these mediators, tryptophan metabolites, SCFAs, and bile acids orchestrate gut–brain axis communication.

The composition of the intestinal microbiota is highly individualized, influenced by a variety of factors, and subject to dynamic changes in response to environmental conditions. Even within a single individual, microbial communities can vary significantly across different regions of the gastrointestinal tract. A healthy gut microbiota is typically characterized by greater taxonomic diversity, enriched genetic content, and a relatively stable core microbial structure. Alterations in the composition or balance of the gut microbiota can influence the initiation and progression of brain tumors by modulating the production and proportion of microbiota-derived metabolites. For example, studies have reported an increased abundance of *Akkermansia muciniphila* in the intestines of glioma patients, which may impact the secretion of related metabolites. Additionally, fecal microbiota sequencing in patients with various brain tumor types has revealed increased levels of Bacteroides, Fusobacteria, and Proteobacteria, alongside decreased abundances of Firmicutes and Actinomycetes, when compared to healthy controls [83]. These microbial shifts may contribute to brain tumor development by altering the metabolic landscape and influencing tumor-associated pathways.

Tryptophan metabolites are synthesized by gut microbiota through either direct tryptophan conversion or via the Kyn pathway. The metabolites facilitate the differentiation and activation of diverse immune cell populations, including M2 macrophages, Treg cells, CD4^+^ T lymphocytes, CD8^+^ T lymphocytes, IL-10/IL-35-producing Bregs, and IL-22-producing group 3 innate lymphoid cells (ILC3s), thereby maintaining intestinal mucosal homeostasis [84]. Additionally, these metabolites promote tumor malignancy and attenuate antitumor immune responses through aryl hydrocarbon receptor (AHR) activation [85]. SCFAs, primarily acetate, butyrate, and propionate, represent major metabolic products of the gut microbiota. These compounds traverse the BBB and modulate glial cell and neuronal function through epigenetic mechanisms [86]. SCFAs modulate innate immune responses through TLR signaling, resulting in activation of NK cells, macrophages, and neutrophils [87]. In adaptive immunity, butyrate predominantly enhances antitumor cytotoxic CD8^+^ T lymphocyte responses via the IL-12 signaling pathway regulated by ID2 activation, ultimately augmenting antitumor therapeutic efficacy [88]. Furthermore, SCFAs regulate immune responses by promoting Treg differentiation and modulating inflammatory processes [80]. Bile acids, including deoxycholic acid, lithocholic acid, acetyldeoxycholic acid, and cholic acid, promote antitumor phenotype adoption in TAMs and enhance the cytotoxic capacity of TILs, thereby potentiating glioma cell elimination [87].

Gut microbiota-derived metabolites additionally demonstrate complex regulatory effects on γδ T cells. SCFAs, particularly propionate, attenuate γδ T cell activity and IL-17 production, while specific commensal microbes promote γδ T cell differentiation. Select phosphorylated microbial metabolites, including (E)-4-hydroxy-3-methyl-but-2-enyl pyrophosphate (HMBPP) and amino bisphosphonates, directly activate γδ T cells, while others mediate their effects through DCs, intercellular interactions, or cytokine signaling networks [89].

### Mechanistic pathways in gut microbiota-mediated effects

**Fig. 3.**
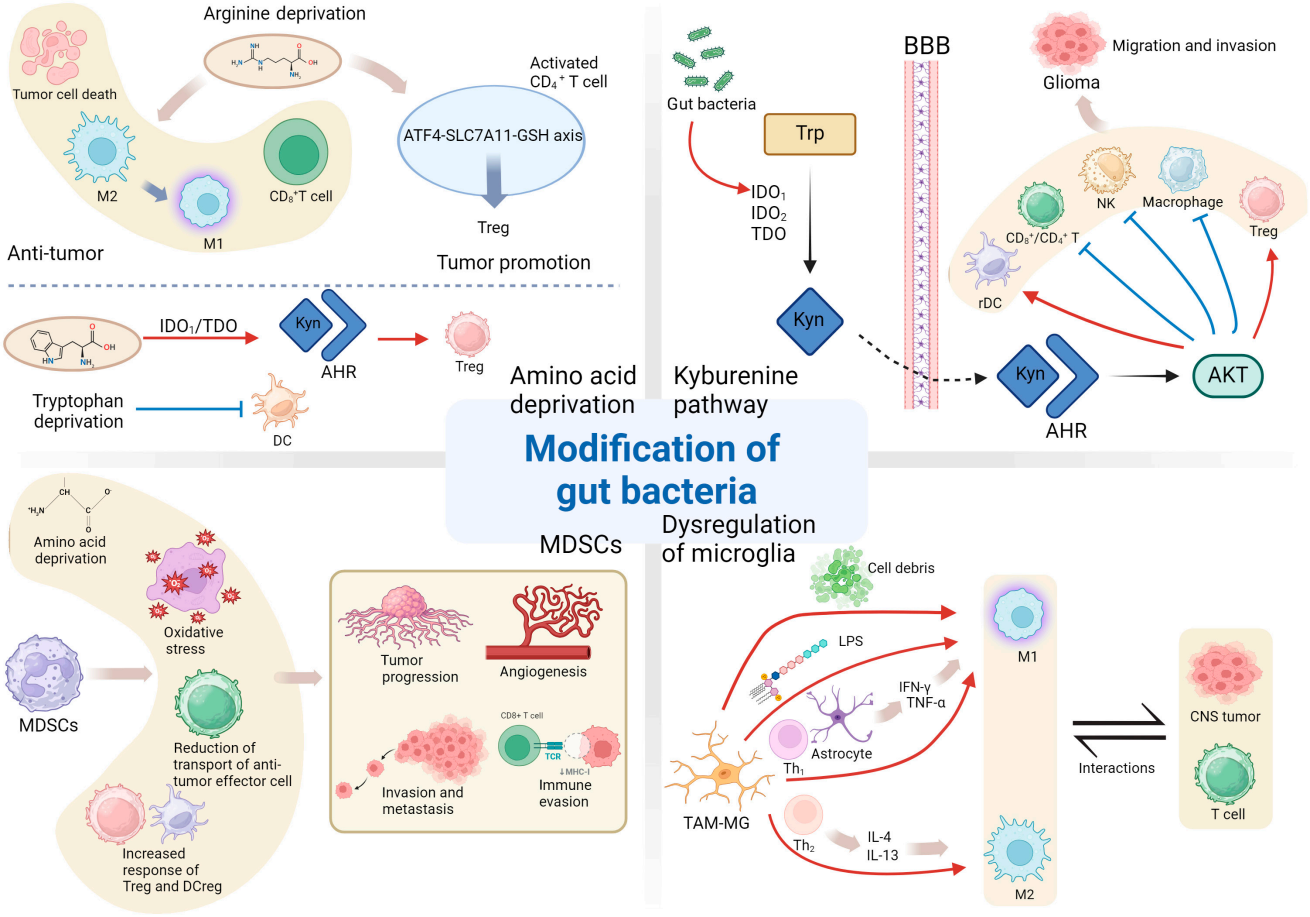
Mechanisms of gut microbiota modification and their impact on CNS neoplasm progression.

Figure 3 illustrates the mechanisms by which gut microbiota modifications influence the TME, highlighting their opposing effects on tumor promotion and suppression through amino acid metabolism, kynurenine pathway activation, dysregulation of microglia, and the activity of MDSCs.

1. Amino acid deprivation (top left):
  o Arginine deprivation: Arginine depletion can have opposing effects. It promotes the reprogramming of M2 microglia/macrophage phenotypes into the pro-inflammatory M1 phenotype, activates CD8^+^ T cells, and enhances antitumor immune response. Conversely, it activates the activating transcription factor 4 (ATF4)–solute carrier family 7 member 11 (SLC7A11)–glutathione (GSH) axis in CD4^+^ T cells, increasing intratumoral Treg accumulation and inducing immunosuppressive effects.
  o Tryptophan deprivation: Tryptophan depletion induces the overexpression of AHR and enhances Kyn uptake, promoting Treg differentiation via IDO1/TDO (tryptophan 2,3-dioxygenase) pathways and inhibiting DC function, contributing to tumor immune evasion.

2. Kyn pathway (top right):
  o Tryptophan metabolism through the Kyn pathway is regulated by IDO1, IDO2, and TDO enzymes, which catalyze the transformation of tryptophan to Kyn. Kyn crosses the BBB and activates AHR, promoting the AHR/Akt pathway, increasing rDCs and Tregs, and decreasing CD8^+^/CD4^+^ T cells, NK cells, and macrophages. This creates an immunosuppressive microenvironment, leading to glioma cell migration and invasion.

3. Dysregulation of microglia (bottom right):
  o Microglia exhibit M1 or M2 polarization. Pro-inflammatory M1 microglia are activated by cell debris, gut-derived compounds [e.g., lipopolysaccharide (LPS)], and cytokines (e.g., TNF-α and IFN-γ) from Th1 cells or astrocytes. In contrast, anti-inflammatory M2 microglia are mediated by Th2-derived IL-4 and IL-13, promoting immunosuppression as well as tissue regeneration. M2 microglia interact with CNS tumors and T cells, contributing to immune evasion.

4. MDSCs (bottom left):
  o MDSCs strongly suppress the immune response via multiple mechanisms such as amino acid deprivation, oxidative stress, reduced transport of antitumor effector cells, and increased responses of Tregs and tolerogenic DCs. These activities promote tumor progression, angiogenesis, invasion, metastasis, and immune evasion.

Red arrows (→) represent activation, promotion, or facilitation of a biological process. Blue T-shaped symbols (⊥) represent inhibition, suppression, or down-regulation of a biological process or function.

The gut microbiota orchestrates its biological effects through multiple pathways, including (a) amino acid metabolism perturbation (specifically arginine and tryptophan), (b) Kyn pathway modulation, (c) microglial homeostasis disruption, and (d) MDSC regulation. These regulatory mechanisms enable the gut microbiota to mediate both oncogenic and oncolytic effects within the CNS [90] (Fig. 3).

Arginine auxotrophy represents a characteristic metabolic feature of GBM [91]. Arginine depletion induces M2-to-M1 microglial repolarization, subsequently activating CD8^+^ T cells and eliciting robust anticancer immunity [92]. Paradoxically, arginine depletion also stimulates CD4^+^ T cells, triggering metabolic and transcriptional remodeling through the ATF4–SLC7A11–GSH axis, culminating in enhanced intratumoral Treg accumulation and immunosuppression [93]. Tryptophan metabolism exhibits heightened activity in brain tumors, particularly gliomas [94], orchestrating immunosuppressive microenvironmental changes through multiple mechanisms that facilitate tumor progression [95]. Tryptophan depletion promotes AHR overexpression and enhances cellular Kyn uptake, thereby facilitating Treg differentiation and mediating IDO1/TDO-induced immunosuppression. Additionally, tryptophan depletion compromises DC function, promoting immune evasion by tumors [96].

The Kyn pathway represents a principal metabolic route for tryptophan catabolism. IDO1, IDO2, and TDO function as critical rate-limiting enzymes in the Kyn pathway [97], catalyzing the conversion of tryptophan to Kyn and its derivatives. Kyn functions as an endogenous ligand for the AHR. IDO1- and TDO2-derived Kyn triggers AHR activation [98], initiating AHR/Akt pathway signaling, resulting in expansion of immunosuppressive DCs and Tregs [99], while suppressing CD8^+^/CD4^+^ T cells, macrophages, and NK cells [100]. This immunosuppressive microenvironment facilitates glial tumor cell ontogeny, multiplication, and metastasis while conferring therapeutic resistance [97]. Significantly, the gut microbiota modulates the Kyn pathway as well as IDO1 activity through regulation of tryptophan accessibility [90]. Gut dysbiosis potentially elevates IDO activity and the Kyn/Trp ratio, thereby accelerating glioma progression.

Microglia-derived tumor-associated macrophages (TAM-MGs) predominantly localize to the tumor periphery [95] and exist in both quiescent and activated states. Activated TAM-MGs exhibit functional plasticity, differentiating into distinct M1 and M2 phenotypes. Multiple factors, including injury-induced cellular debris, gut microbiota-derived compounds (particularly LPS), and T helper 1 (Th1)/astrocyte-derived cytokines (notably IFN-γ and TNF-α), promote M1 phenotype polarization, initiating inflammatory responses. Conversely, cytokines IL-4 and IL-13 derived from Th2 promote M2 phenotype polarization, orchestrating immunosuppression and tissue repair processes [101]. In GBM, microglia undergo severe oxidative stress, activating the NR4A2–SQLE axis and consequently enhancing tumor proliferation [102]. Within CNS tumors, microglia establish complex interactions with neoplastic cells as well as infiltrating peripheral immunocytes, fundamentally reshaping the TME. In low-grade gliomas (LGGs), cancer stem cells secrete CX3CL1 to facilitate microglial recruitment, which subsequently promotes tumor growth through CCL5 secretion. Anti-inflammatory microglia elevate platelet-derived growth factor receptor β (PDGFRB) expression levels in glioma cells, a phenomenon particularly prevalent in HGGs. In sonic hedgehog-activated medulloblastoma mouse models, tumor cells undergo transdifferentiation into astrocyte-like cells, synthesizing IL-4 that activates TAM-MGs. Activated TAM-MGs produce insulin-like growth factor 1 (IGF1), which functions through autocrine/paracrine signaling to regulate the development and progression of pediatric ependymomas. In microglia–T cell interactions, CX3CR1-dependent CXCL10 expression mediates microglial recruitment to the tumor site, where they implement T cell suppression through multiple immune checkpoints, including PD-1, cytotoxic T lymphocyte-associated protein 4 (CTLA-4), and forkhead box P3 (Foxp3). Attenuation of TAM-MG-mediated T cell suppression requires down-regulation of V-domain immunoglobulin suppressor of T cell activation (VISTA) and PD-L1 signaling pathways. Within GBM, mechanistic target of rapamycin (mTOR) signaling functions as a critical regulator of TAM-MG immunosuppressive phenotype acquisition. Collectively, microglia undergo phenotypic transformation in the TME, adopting immunosuppressive characteristics that promote tumor growth [103].

MDSCs represent a heterogeneous population of pathologically activated immature monocytes and neutrophils. MDSCs exhibit potent immunosuppressive capabilities and orchestrate multiple processes in tumor biology, involving malignant progression, angiogenesis, invasion, metastasis, and immune escape, thereby correlating with poor clinical outcomes [104,105]. MDSCs modulate the immune microenvironment through multiple mechanisms, including amino acid metabolism disruption, oxidative stress induction, impairment of antitumor effector cell trafficking, and enhancement of Treg and DC responses [105]. MDSCs suppress antigen-specific CD8^+^ T cell function predominantly through elevated expression of ARG1, inducible nitric oxide synthase (iNOS), and reactive oxygen species (ROS) [106].

### Regulatory mechanisms of gut microbiota in programmed cell death

**Fig. 4.**
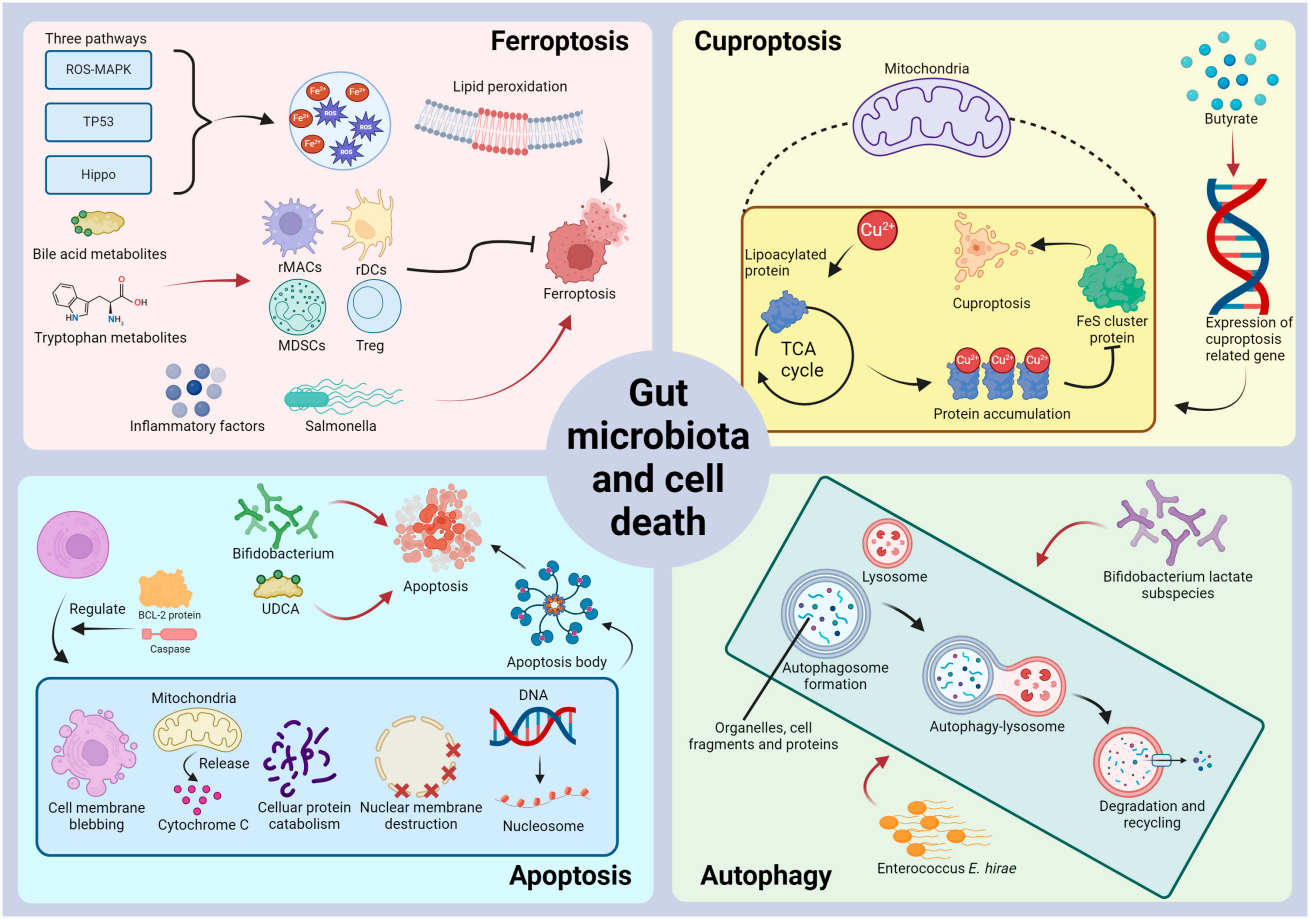
Gut microbiota and its influence on various forms of cell death.

Figure 4 illustrates the roles of gut microbiota and their derived metabolites in mediating or influencing 4 principal types of cell death—ferroptosis, cuproptosis, apoptosis, and autophagy—and their implications in tumor progression, immunity, therapy, and prognosis.

1. Ferroptosis (top left):
  o Ferroptosis is triggered by iron accumulation, lipid peroxidation, and excessive ROS, leading to cell membrane damage and rupture. Key signaling pathways include ROS–MAPK, TP53, and Hippo.
  o Metabolites from intestinal flora, especially bile acid and tryptophan metabolites, facilitate the differentiation and function of immunosuppressive cells, including rMacs, rDCs, MDSCs, and Tregs, which collectively inhibit ferroptosis.
  o In contrast, inflammatory factors derived from gut microbiota and certain bacteria, such as *Salmonella*, induce ferroptosis in tumor cells.

2. Cuproptosis (top right):
  o Cuproptosis is initiated by abnormal accumulation of copper ions (Cu^2+^), which interact with lipoacylated proteins in the tricarboxylic acid (TCA) cycle, leading to protein accumulation, proteotoxic stress, and cell dysfunction.
  o Gut microbiota-derived butyrate enhances the expression of cuproptosis-related genes, potentially regulating tumor progression by influencing this form of cell death.
  o Apoptosis (bottom left):
  o Apoptosis is characterized by cell membrane blebbing, cytochrome c release from mitochondria, nuclear membrane destruction, protein catabolism, and DNA fragmentation into nucleosomes.
  o Gut microbiota, such as *Bifidobacterium*, and microbial metabolites, such as ursodeoxycholic acid (UDCA), induce apoptosis through regulation of apoptosis-related proteins, involving caspases and BCL-2.

3. Autophagy (bottom right):
  o Autophagy is a degradation pathway mediated by lysosome, involving autophagosome formation, their fusion with lysosomes, and the recycling of cellular components.
  o Microbial species, such as *E. hirae* and *Bifidobacterium lactate* subspecies, promote autophagy by facilitating autophagosome formation and lysosomal degradation.

Red arrows (→) represent activation, promotion, or facilitation of a biological process.

Diverse types of programmed cell death, involving ferroptosis, copper-dependent cell death, apoptosis, and autophagy, contribute critically to the maintenance of cellular homeostasis (Table 4). Accumulating evidence demonstrates that gut microbiota and their metabolites extensively modulate these cell death pathways, profoundly affecting tumor initiation, progression, immune responses, therapeutic outcomes, and clinical prognosis.

**Table 4.** The forms, mechanisms, relevant bacterial microbiota, and their metabolites of cell death

Cell death form	Related mechanism	The associated gut microbiota and their metabolites	Reference
Ferroptosis	Iron accumulation increases, lipid peroxidation, and excessive generation of ROS cause cell membrane damage, rupture, and death	Bile acid metabolites Tryptophan metabolites Gut microbiota inflammatory factors Salmonella	[107,108,110,111]
Cuproptosis	Copper ions abnormally accumulate in cells and interact with acylase enzymes in the tricarboxylic acid cycle, causing protein accumulation, protein toxicity stress, impaired cellular function, and eventual cell death	Butyrate	[112–114]
Apoptosis	Cytochrome c is released by mitochondria, the nuclear membrane is disrupted, proteins inside the cell undergo extensive cleavage, vesicles and genomic DNA break down into nucleosomal structures, while the integrity of the cytoplasmic membrane is preserved	Bifidobacterium Ursodeoxycholic acid	[115–118]
Autophagy	Depend on lysosomal protein degradation	*Enterococcus E. hirae* *Lactobacillus* subsp. *SF*	[119–124]

Ferroptosis is an iron-dependent, non-apoptotic form of cell death characterized by intracellular iron accumulation, lipid peroxidation, and excessive ROS production, ultimately resulting in cell membrane damage and rupture. Multiple molecular mechanisms regulate ferroptosis, including ROS–MAPK (mitogen-activated protein kinase), TP53, and Hippo signaling pathways, while key subcellular organelles such as lysosomes, mitochondria, and endoplasmic reticulum play critical roles in the execution and regulation of this process [107–109]. Gut microbiota-mediated ferroptosis orchestrates tumorigenesis, disease progression, and immunotherapeutic responses through extensive reprogramming of TME immune cells. Gut microbiota-derived metabolites, including both immunosuppressive and inflammatory mediators, modulate ferroptosis through distinct effects on immune cell differentiation and function. Specifically, immunosuppressive metabolites, particularly bile acid and tryptophan derivatives, facilitate the differentiation and activation of immunoregulatory cells—including regulatory macrophages (rMacs), rDCs, MDSCs, and Tregs—thereby attenuating ferroptotic processes. Conversely, inflammatory metabolites, notably microbiota-derived inflammatory cytokines, potentiate ferroptotic cell death [110]. The gut microbiota directly mediates ferroptosis, as exemplified by *Salmonella*-induced suppression of GPX4 expression, resulting in ferroptotic cell death and subsequent inhibition of glioma progression [111].

Cuprotosis represents a distinct type of programmed cell death featured by copper ion dysregulation. This process is mechanistically characterized by aberrant intracellular copper ion accumulation, which disrupts acyltransferase function in the TCA cycle, triggering a cascade of events including protein aggregation, proteotoxic stress, cellular dysfunction, and eventual cell death [112]. The extent of copper-dependent cell death demonstrates a positive correlation with macrophage infiltration, simultaneously promoting immune activation while facilitating immune escape mechanisms. Consequently, quantification of copper-dependent cell death signatures could be valuable prognostic indicators in glioma and predict immunotherapy efficacy, with elevated levels correlating with poor clinical outcomes [113]. Evidence indicating that the gut microbiota metabolite butyrate up-regulates copper-dependent cell death-related genes suggests that the intestinal flora and its metabolites may orchestrate tumor immunity and influence disease progression through modulation of copper-dependent cell death pathways [114].

Apoptosis represents a highly orchestrated form of programmed cell death governed by BCL-2 proteins and caspases, featured by sequential events including mitochondrial cytochrome c release, nuclear envelope breakdown, systematic protein degradation, membrane blebbing, and nucleosomal DNA fragmentation, while preserving plasma membrane integrity. Apoptosis proceeds through 2 distinct pathways—extrinsic and intrinsic—distinguished by their initiating stimuli and differential engagement of death receptors [115,116]. Emerging evidence demonstrates that gut microbiota and their metabolites serve as critical regulators of apoptotic processes. Specifically, the probiotic *Bifidobacterium* exhibits antineoplastic properties through dual mechanisms: induction of mitochondria-mediated apoptosis and suppression of growth factor signaling pathways [117]. The gut microbiota-derived metabolite ursodeoxycholic acid (UDCA) triggers endoplasmic reticulum stress-associated apoptosis, thereby attenuating GBM progression and exhibiting therapeutic potential [118].

Autophagy constitutes a lysosome-dependent protein degradation pathway, with autophagic cell death representing a unique type of non-apoptotic programmed cell death [119,120]. Autophagy exhibits context-dependent functions: During early tumorigenesis, it suppresses tumor initiation and progression; however, in advanced stages, it facilitates tumor survival, metastasis, and invasion [121]. In gliomas, both cytotoxic and cytoprotective autophagy critically influence tumorigenesis, therapeutic resistance, and cellular differentiation. Furthermore, CNS cancer stem cells frequently display dysregulated autophagic flux [120]. The gut microbiota exerts multifaceted effects on autophagy, thereby modulating tumor development, progression, and therapeutic responses. *Enterococcus hirae* induces autophagy in intestinal cells through enhanced local dopamine signaling while concurrently stimulating immune responses and restructuring the host microbiome, collectively enhancing therapeutic efficacy [122]. *Bifidobacterium animalis* subsp. *lactis* SF potentiates tumor apoptosis and autophagy through multiple mechanisms: attenuation of intestinal inflammation, reduction of TGF-β translocation, and suppression of phosphatidylinositol 3-kinase (PI3K)/Akt signaling, ultimately augmenting irinotecan efficacy [123]. Comparative studies between GF and conventional mice demonstrated that Taohong Siwu Decoction administration resulted in enhanced autophagy and suppressed glioma cell proliferation, indicating gut microbiota-dependent modulation of its therapeutic efficacy [124].

Table 4 summarizes the relationship between different cell death forms and gut microbiota. Ferroptosis is driven by iron accumulation and lipid peroxidation, influenced by bile acid and tryptophan metabolites, as well as inflammatory factors from gut bacteria like *Salmonella*. Cuproptosis results from copper ion accumulation disrupting cellular functions, with butyrate as a related microbial metabolite, while apoptosis and autophagy are regulated by specific gut bacteria such as *Bifidobacterium*, *Enterococcus E. hirae*, and *Lactobacillus* subsp. *SF.*

## Advanced Cancer Immunotherapy and Molecular-Targeted Therapeutic Approaches

**Fig. 5.**
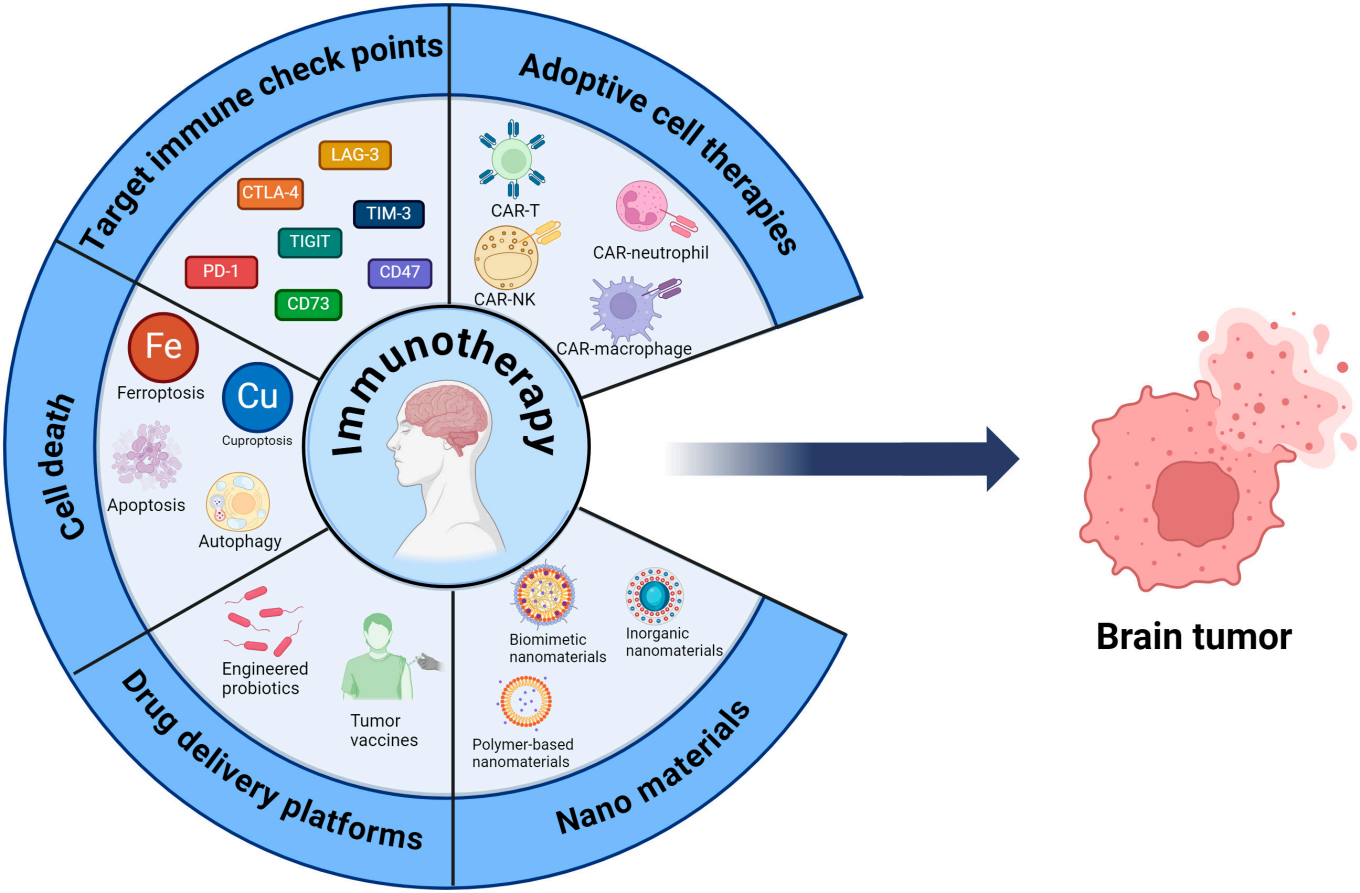
Advanced therapeutic strategies for brain tumors: immunotherapy and targeted therapy.

Figure 5 illustrates the major approaches in advanced therapeutic strategies targeting brain tumors, focusing on immunotherapy and targeted therapies. Immunotherapy strategies include the therapeutic modulation of immune checkpoints (e.g., PD-1/PD-L1, CTLA-4, TIM-3, LAG-3, TIGIT, CD47, and CD73) and adoptive cell therapy with CAR-modified immunocytes, especially CAR-T cells, CAR-neutrophils, CAR-NK cells, and CAR-macrophages. Regulated and programmed cell death pathways—ferroptosis, cuproptosis, apoptosis, and autophagy—are also explored as therapeutic targets. Advanced drug delivery systems, including engineered probiotics and bacteria-based tumor vaccines, enhance therapeutic precision. Nanomaterial-based platforms, such as polymer-based, biomimetic, and inorganic nanomaterials, provide innovative solutions for targeted delivery and controlled release of therapeutic agents. The approaches are collectively intended to boost therapeutic effect and overcome the challenges in brain tumor treatment.

### Definition of immunotherapy

Immunotherapy has revolutionized cancer treatment by restoring and enhancing endogenous antitumor immunity through multi-level modulation of the immune response. Current strategies include immune checkpoint blockade, adoptive cell therapy, oncolytic virotherapy, cancer vaccines, and cytokine therapies, all of which have shown substantial efficacy across a range of malignancies and driven major advances in tumor immunology [125,126].

Importantly, the gut–brain axis has emerged as a critical regulator of immunotherapy outcomes. Through microbiota-mediated signaling, neuroimmune modulation, and systemic immune priming, this bidirectional communication network influences the activation of immune cells, the remodeling of the TME, and therapeutic responsiveness. Understanding and targeting the gut–brain–immune axis thus offers new opportunities to enhance the efficacy of cancer immunotherapies [127].

#### Therapeutic modulation of immune cells: Engineering CAR-based cellular therapeutics

Tumor-infiltrating immune cells within the CNS TME exert dual roles in tumor progression and immune surveillance, forming the basis for immune cell-based therapeutic strategies [128]. Among these, chimeric antigen receptor (CAR)-modified immune cells—including T cells, NK cells, neutrophils, and macrophages—have emerged as transformative platforms for CNS malignancies.

CAR-T cells, engineered to recognize tumor-associated antigens (TAAs) such as IL13Rα2, HER2, epidermal growth factor receptor (EGFR)/EGFRvIII, and B7-H3, have shown promise in GBM and pediatric brain tumors [129,130]. However, their efficacy is limited by intratumoral heterogeneity, TME immunosuppression, and delivery challenges [130]. Intrathecal delivery of CAR-T cells targeting HER2, EPHA2, and IL13Rα2—alone or with azacitidine, has shown encouraging outcomes in medulloblastoma and ependymoma [131]. Success in clinical translation demands optimization of delivery, T cell persistence, therapeutic windows, multi-targeting strategies, and synergy with standard treatments [132]. CAR-neutrophils, enabled by CRISPR/Cas9 editing, overcome the BBB and deliver TME-responsive nanotherapeutics to GBM, despite inherent challenges such as short lifespan and low transfection efficiency [133]. CAR-NK cells exert both direct cytotoxicity and indirect immunomodulation via DC support. Although endogenous NK cells are suppressed in GBM, cytokine activation and CAR engineering restore functionality and tumor specificity, supporting their therapeutic application [134]. CAR-macrophages, engineered in situ via DNA nanocarriers (such as ErbB2-specific CARs), enhance phagocytic clearance of tumor cells, offering a novel approach particularly effective in brainstem glioma [135].

#### Targeting immune checkpoints

Immune checkpoints are key regulators of immune activity that can be co-opted by CNS tumors to enable immune evasion. Therapeutic blockade of checkpoint molecules—such as PD-1/PD-L1, CTLA-4, TIM-3, LAG-3, TIGIT, CD47, and CD73—aims to reverse immunosuppression and restore antitumor immunity [127,136,137].

**Fig. 6.**
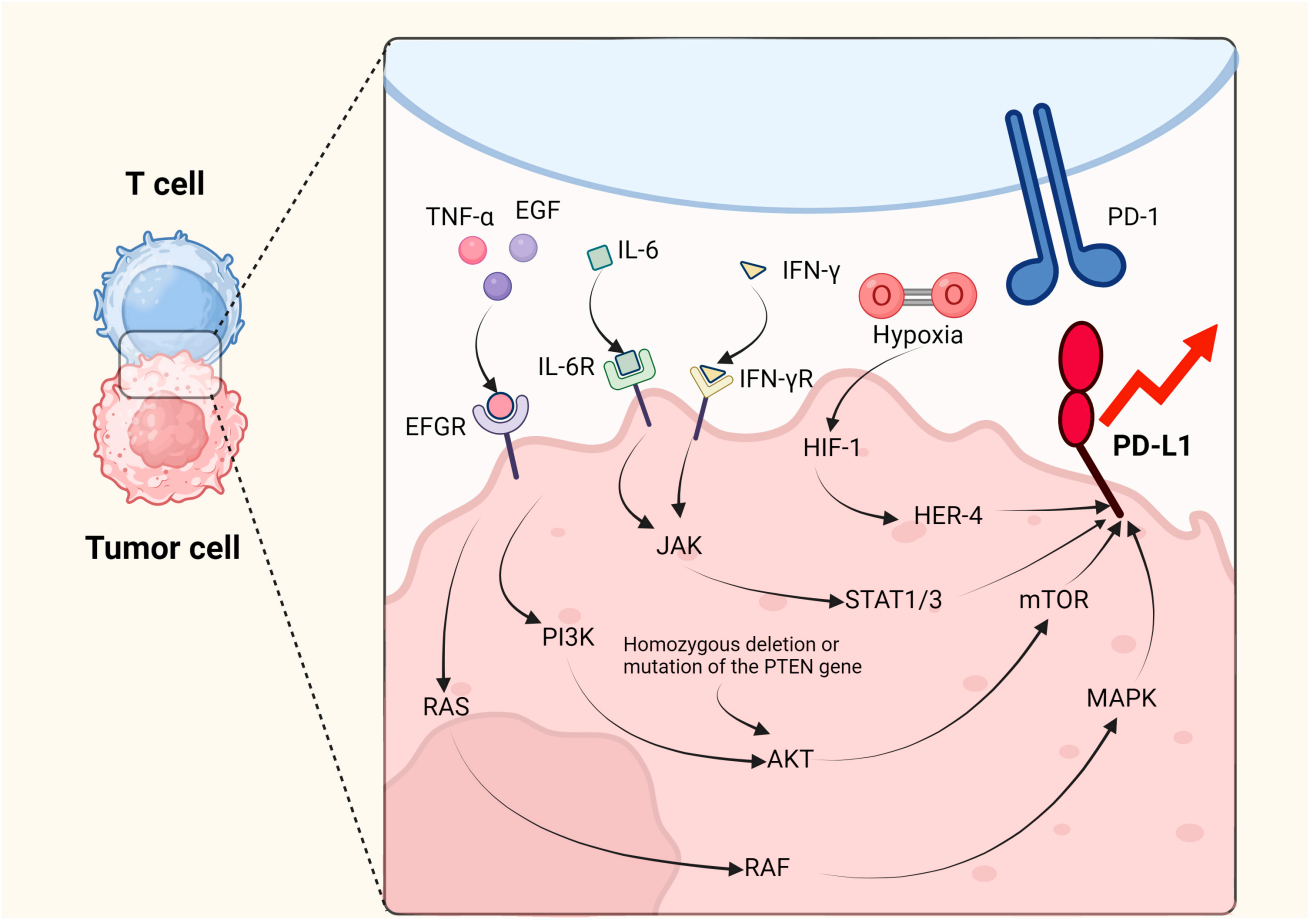
Molecular mechanisms governing PD-L1 expression and PD-1/PD-L1 axis-mediated immune escape in the TME.

Among them, the PD-1/PD-L1 axis plays a central role in GBM by suppressing T cell function and promoting an immunosuppressive microenvironment. PD-1 is widely expressed on T cells, NK cells, macrophages, B cells, DCs, and other myeloid cells, while PD-L1 is up-regulated on tumor and antigen-presenting cells (APCs) via multiple oncogenic and cytokine-driven pathways. Key pathways and regulators of PD-L1 expression include EGFR/PI3K/Akt/mTOR and Ras/RAF/MAPK, activated by TNF-α and EGF; PTEN loss, which enhances Akt signaling; IFN-γ, via IFN-γR–Janus kinase (JAK)/signal transducer and activator of transcription (STAT) signaling; IL-6, through JAK/STAT3 pathway activation; and hypoxia, via HIF-1α stabilization (Fig. 6). Within the GBM TME, PD-1/PD-L1 signaling contributes to CD8^+^ T cell dysfunction and apoptosis, CD4^+^ T cell exhaustion and cytokine dysregulation, expansion of Tregs and Bregs, M1-to-M2 macrophage polarization, and MDSC-mediated immunosuppression.

Collectively, these mechanisms foster an immunosuppressive milieu that promotes tumor progression and presents critical targets for checkpoint-based immunotherapy [138].

The engagement of T cell-expressed PD-1 with tumor cell-derived PD-L1 orchestrates immunosuppressive signaling cascades, thereby promoting tumor immune escape mechanisms. Multiple pathways up-regulate PD-L1 expression.

• EGFR signaling: TNF-α and EGF activate EGFR, which initiates the Ras/RAF/MAPK and PI3K/Akt/mTOR signaling pathways, leading to increased PD-L1 expression.
• PTEN mutations: Homozygous mutation or loss of the PTEN gene enhances PI3K/Akt/mTOR signaling, promoting Akt activation and up-regulating PD-L1 expression.
• IFN-γ: Engagement of cell surface IFN-γR receptors induces PD-L1 expression.
• IL-6 signaling: IL-6 triggers the JAK/STAT pathway, leading to STAT phosphorylation and subsequent PD-L1 up-regulation.
• Hypoxia: Hypoxic conditions enhance PD-L1 expression through HIF-1.

**Fig. 7.**
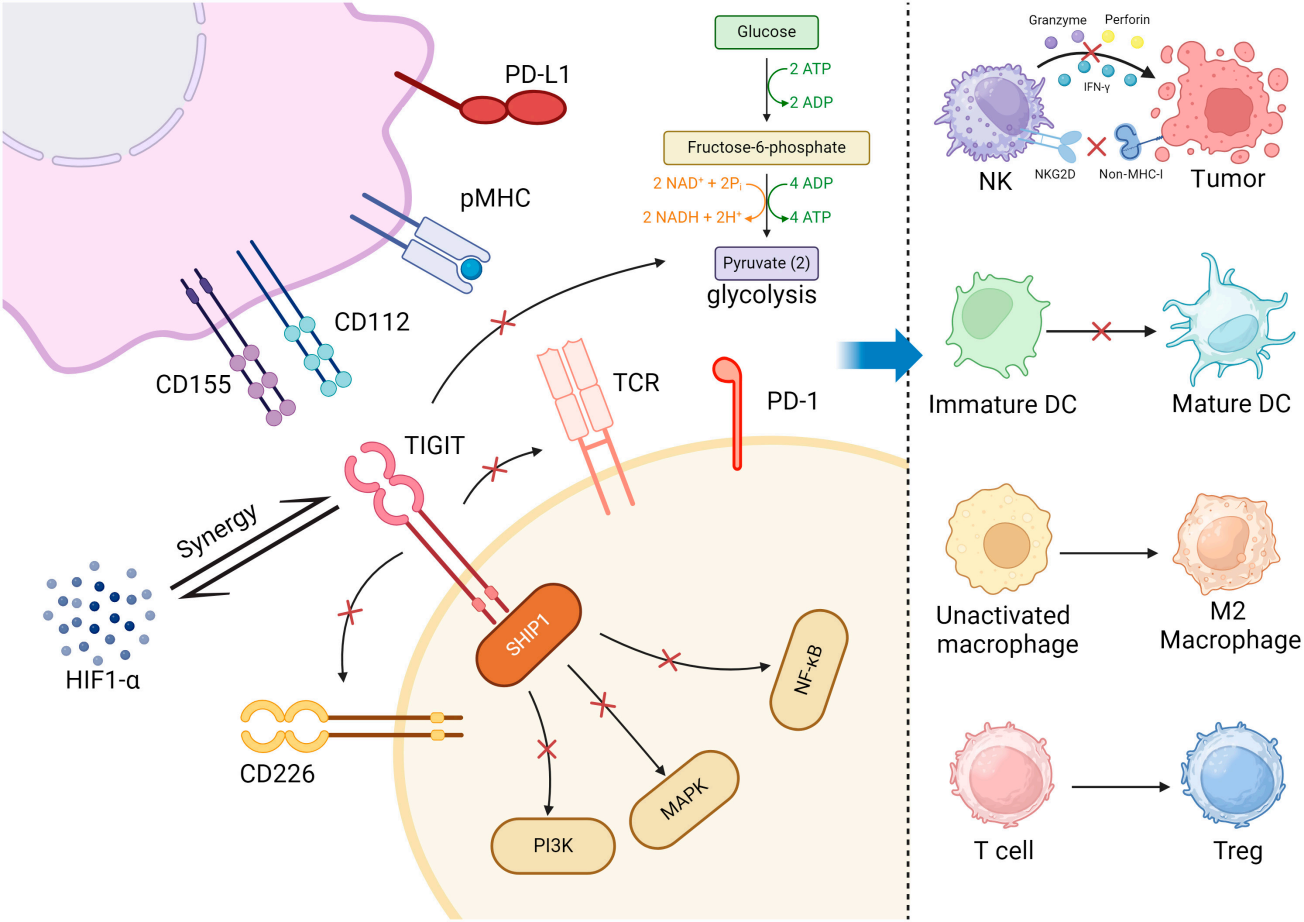
TIGIT-mediated immunosuppressive mechanisms in the TME.

Beyond PD-1/PD-L1, several inhibitory immune checkpoints, including CTLA-4, LAG-3, TIM-3, and TIGIT, play pivotal roles in modulating antitumor immunity, particularly in CNS tumors [139] (Fig. 7).

CTLA-4, expressed on activated T cells, binds B7 molecules on APCs to suppress CD28-mediated costimulation. It transduces inhibitory signals via SYP/p52SHC and PI3K/Akt pathways, leading to Treg expansion, inhibition of B and NK cell functions, and broad immunosuppression that promotes tumor immune evasion [140,141].

LAG-3 is highly expressed on exhausted T cells and subsets of Tregs [142]. Through interaction with ligands such as MHC class II, FGL1, and galectin-3, LAG-3 disrupts T cell receptor (TCR) signaling by limiting ZAP70 phosphorylation, uncoupling Lck from CD4^+^ and CD8^+^ T cell co-receptors, and altering synaptic signaling. It also undergoes extracellular cleavage via ADAM10/17. These mechanisms collectively drive T cell dysfunction and immune exhaustion [143].

TIM-3 is predominantly expressed on TAMs in gliomas and correlates with poor clinical prognosis [144]. It modulates multiple innate immune pathways, including TLR2/4, TLR7/9, cGAS–STING, and the NLRP3 and NLRC4 inflammasomes. These cascades contribute to T cell exhaustion, M2 macrophage polarization, NK cell dysfunction, and impaired DC maturation, thereby reinforcing an immunosuppressive TME [145].

TIGIT is broadly expressed on CD4^+^ and CD8^+^ T cells, Tregs, NK cells, and TILs [146]. It competes with CD226 for binding to CD155 and CD112, thereby attenuating costimulatory signaling. TIGIT also suppresses glycolytic metabolism, cooperates with HIF-1α under hypoxic conditions, and recruits SHIP1 to inhibit NF-κB, MAPK, and PI3K pathways. These actions lead to impaired NK cytotoxicity, DC maturation blockade, M2 macrophage skewing, and Treg induction. Notably, TIGIT is often coexpressed with PD-1, and their dual blockade exhibits synergistic therapeutic effects [147].

Figure 7 depicts the mechanisms through which TIGIT mediates immune suppression within the TME, divided into 2 sections:

• Left panel: TIGIT competes with CD226 for binding to CD155 and CD112, impairing CD226 activation and function. It down-regulates the expression of the TCR complex, blocks glycolysis, and synergizes with HIF-1α. Upon binding to CD155, TIGIT recruits SHIP1 to suppress the PI3K, MAPK, and NF-κB signaling pathways, further enhancing its immunosuppressive effects.
• Right panel: Through these mechanisms, TIGIT suppresses NK cell cytotoxicity, suppresses the maturation of DCs, promotes M2 macrophage differentiation, and induces T cell differentiation into Tregs, thereby facilitating tumor immune evasion.

**Fig. 8.**
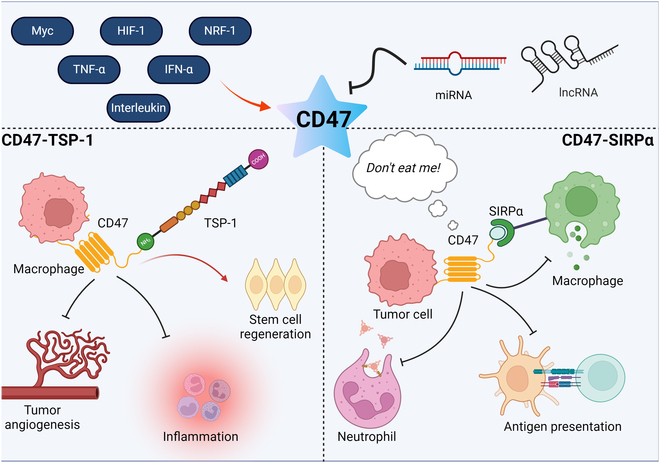
Regulation and functional roles of CD47 in tumor immunity.

CD47 is an immunoregulatory checkpoint molecule highly expressed on tumor cells and regulated through diverse mechanisms. Its expression is transcriptionally activated by Myc, HIF-1, and NRF-1, up-regulated by proinflammatory cytokines such as TNF-α and IFN-γ, and negatively regulated posttranscriptionally by microRNAs and long noncoding RNAs (Fig. 8).

CD47 binds 2 principal ligands, thrombospondin-1 (TSP-1) and signal regulatory protein α (SIRPα), mediating distinct immunological and biological outcomes. The CD47–TSP-1 axis suppresses angiogenesis via VEGFR2 inhibition, thereby limiting tumor growth and metastasis, modulating inflammatory responses, and promoting tissue regeneration. More critically, CD47 interaction with SIRPα on myeloid cells initiates a “don’t-eat-me” signal that enables tumor cells to evade phagocytosis by macrophages and neutrophils. This axis also impairs APC function, dampening T cell-mediated immune responses and contributing to tumor immune escape [148].

CD47 is regulated at multiple levels. Its expression is up-regulated through transcription factors, particularly Myc, HIF-1, and NRF-1, as well as cytokines, including TNF-α, IFN-γ, and ILs. Conversely, microRNAs (miRNAs) and long noncoding RNAs (lncRNAs) attenuate CD47 expression. CD47 interacts with 2 primary ligands: TSP-1 and SIRPα. The CD47–TSP-1 interaction inhibits tumor angiogenesis, reduces inflammation, and promotes stem cell regeneration. On the other hand, the CD47–SIRPα binding transmits a “don’t eat me” signal, preventing phagocytosis of tumor cells by neutrophils and macrophages. Additionally, CD47–SIRPα signaling may impair APC function, causing T lymphocyte dysfunction and promoting immune evasion. Red arrows indicate activation, promotion, or up-regulation of a biological process. Inhibition symbols (T-shaped lines) indicate suppression, inhibition, or down-regulation of a biological process.

Ecto-5′-nucleotidase (CD73) is a highly expressed immunoregulatory checkpoint on tumor cells and a key enzyme in purinergic signaling. By converting extracellular adenosine triphosphate (ATP) to adenosine, CD73 contributes to immunosuppressive reprogramming of the TME. The accumulated adenosine engages A2A and A2B receptors on immune cells, broadly suppressing T cell function, skewing DCs toward a tolerogenic phenotype, impairing NK cell development and activity, and modulating the responses of Tregs, macrophages, and neutrophils. Beyond immune suppression, the CD73–adenosine axis directly promotes tumor progression by enhancing cancer cell proliferation, invasiveness, and metastatic potential. It also stimulates angiogenesis and drives EMT, further supporting tumor growth and dissemination [149].

Table 5 summarizes key immune checkpoints, their expression sites, related signaling pathways, and their roles in the tumor microenvironment. Immune checkpoints like PD-1, CTLA-4, LAG-3, and TIM-3 primarily contribute to T cell exhaustion, immune suppression, and the promotion of regulatory cell populations. Other checkpoints, such as CD47 and CD73, help tumor cells evade immune detection by inhibiting phagocytosis, suppressing antigen-presenting cells, and altering immune cell functions.

**Table 5.** Immune checkpoint types, expression sites, relevant mechanistic pathways, and functions

Types of immune checkpoints	Site of expression	Relevant mechanism or pathway	Function in the tumor microenvironment	Reference
PD-1	T lymphocytes, monocytes–macrophages, B lymphocytes, DCs, myeloid cells, and NK cells	Ras/RAF/MAPK PI3K/Akt/mTOR IFN-γ/IFN-γR JAK/STAT PD-1/PD-L1	Induce exhaustion of CD4^+^ and CD8^+^ T cells, induce differentiation of T cells and B cells into regulatory cells, induce early M1 polarization of GAMs and followed by late M2 polarization	[139]
CTLA-4	Activate T cells	CTLA-4/B7 CTLA-4/SYP/p52SHC CTLA-4/PI3K/Akt	Inhibit activation and multiplication of Tregs, enhance generation and survival of Tregs, inhibit activation of B cells and antibody production, reduce NK cell degranulation activity, and suppress secretion of IFN-γ	[141,142]
LAG-3	Macrophages	LAG3/MHC class II LAG3/LSECtin LAG3/GAL-3 LAG3/FGL1 LAG3/TCR-CD3 LAG3/prefoldin-α LAG3/synuclein fibrils	Destroy T cell function and induce their exhaustion	[143,144]
TIM-3	Depleted T cells and numerous Tregs	Interfere with TLR2/4 and NF-κB, TLR7/9, cGAS–STING, NLRP3, and NLRC4 inflammasomes	Induce T cell exhaustion and dysfunction, promote macrophage M2 polarization, activate or deplete NK cells, inhibit DC maturation and function	[145,146]
TIGIT	CD4^+^ T cells, CD8^+^ T cells, NK cells, Tregs, and TILs	Binding to CD155 recruits SHIP1 to inhibit the PI3K, MAPK, and NF-κB pathways	Inhibit NK cell cytotoxic activity, suppress DC maturation, promote macrophage M2 polarization, induce T cell differentiation into Tregs	[147,148]
CD47	Tumor cells	CD47/TSP-1 CD47/SIRPα	Inhibit angiogenesis, suppress the phagocytosis of tumor cells by macrophages and neutrophils, inhibit the function of APC leading to T lymphocyte dysfunction	[149]
CD73	Tumor cells	CD73/A_2A_ or A_2B_ adenosine receptors	Inhibit T cell function, induce DC toward a pro-tumor phenotype, suppress NK cell maturation and function, regulate Tregs, macrophages, and neutrophils	[150]

#### Therapeutic development of immune checkpoint-targeting antibodies and blocking agents

Extensive profiling of immune checkpoints in CNS malignancies has prompted the development of targeted antibodies and blocking agents. However, monotherapy with PD-1/PD-L1 inhibitors has demonstrated limited efficacy in GBM, largely due to the immunosuppressive TME [150,151]. To overcome this, combination strategies have shown promising results.

Metabolic targeting, such as inhibition of hexokinase 2 (HK2) or GLUT1, synergizes with PD-1 blockade by reversing immune evasion and T cell exhaustion [152]. Similarly, Chek2 inhibition enhances antigen presentation and STING activation, improving responsiveness to PD-1 therapy [153]. Targeting immune checkpoints expressed on myeloid cells, including Siglec-9 and TREM2, reprograms macrophages and augments T cell infiltration [154]. Other approaches, including ELTD1 depletion, IL-8/CXCR1/2 axis blockade, and chlorogenic acid co-administration, remodel the TME to potentiate PD-1 inhibition [155–157]. Innovative delivery systems such as injectable thermogels and intranasal small interfering RNA (siRNA), as well as gut microbiota modulation (e.g., high-glucose diet), further enhance therapeutic outcomes [158,159].

Novel modalities like long half-life IL-2, intratumoral DNX-2401, low-intensity pulsed ultrasound (LIPU) with microbubbles, and bacterial photothermal therapy have demonstrated the ability to enhance CNS penetration and anti-PD-1 efficacy [160–163]. STING pathway agonists (e.g., β-mangostin) and IL-12-secreting mesenchymal stem cells further promote cytotoxic T cell responses and M1 polarization, establishing durable antitumor immunity [164,165]. Losartan, by mitigating cerebral edema, also improves PD-1 blockade outcomes [166].

CTLA-4 targeting has emerged as a complementary strategy. Nanoparticle (NP)-based delivery of anti-CTLA-4 antibodies enhances BBB penetration and local immune activation. Fc-enhanced anti-CTLA-4 antibodies (e.g., botensilimab) achieve selective Treg depletion in the TME while preserving peripheral tolerance, synergizing with PD-1 inhibitors [167,168].

Second-generation checkpoints, including TIM-3, LAG-3, and TIGIT, are gaining attention. TIM-3 blockade has shown efficacy in diffuse midline glioma [169]. TIGIT-targeted therapies, including synNotch-programmed NK cells and anti-TIGIT/PD-1 dual blockade, reverse MDSC-mediated immunosuppression and promote T cell infiltration [142,170]. While LAG-3 remains a promising target, effective therapeutic agents are still in development.

CD47 blockade represents a compelling approach to overcome phagocytic suppression in GBM. Hydrogel-based local delivery of anti-CD47 with TMZ enhances macrophage and NK cell activation [171]. Targeting CD47 phosphorylation (via EGFR/c-SRC), radiosensitization strategies, and CI-994 co-administration significantly improve therapeutic outcomes [172–174]. Focused ultrasound-mediated BBB opening further enhances delivery efficacy [175]. Similar benefits have been observed in meningioma models [176].

CD73 inhibition disrupts adenosine-mediated immunosuppression. NPs down-regulating CD73 expression improve T cell activation and show synergy with radiotherapy [177]. Intranasal delivery of CD73 siRNA has demonstrated TME remodeling and tumor regression in preclinical glioma models [178].

Collectively, these strategies underscore the necessity of combinatorial and multimodal approaches targeting metabolic pathways, novel checkpoints, and TME components to optimize immune checkpoint therapy in CNS tumors. These emerging combination immunotherapies for CNS tumors may be further enhanced by modulation of the gut–brain axis, which influences systemic immunity, T cell function, and the TME via microbial metabolites and immune signaling. Thus, targeting the gut microbiota offers a promising avenue to synergize with immune checkpoint blockade and improve therapeutic outcomes in brain tumors.

### Programmed and regulated cell death mechanisms

Multiple regulated cell death (RCD) pathways, including ferroptosis, cuproptosis, apoptosis, and autophagy, play essential roles in the pathogenesis and therapy resistance of CNS malignancies, particularly gliomas. Therapeutic modulation of these pathways offers a promising strategy to overcome the challenges of tumor recurrence, invasion, and resistance.

Ferroptosis, an iron-dependent lipid peroxidation-driven cell death mechanism, has emerged as a dominant RCD form in gliomas. Inhibition of GPX4 induces immunogenic ferroptosis, enabling the development of ferroptotic cancer vaccines [179]. Engineered NPs and pharmacological agents targeting GPX4, SLC7A11, NQO1, or SOAT1 have been shown to enhance ferroptotic sensitivity and potentiate radiotherapy or immunotherapy [180,181]. Strategies such as cysteine/methionine deprivation, TRIM7 or PRR11 knockdown, and Hsp90-Acsl4 or SIRT3 axis modulation have further demonstrated synergy with conventional treatments [182–186]. Ferroptosis not only promotes tumor regression but also reprograms the tumor immune microenvironment, rendering resistant GBMs more susceptible to TMZ [187].

Cuproptosis, a copper-dependent form of cell death, is gaining recognition as a novel glioma vulnerability. Copper overload or disrupted copper homeostasis, via agents like elesclomol, Cu-based nanozymes, or regorafenib-based nanoplatforms, induces mitochondrial protein aggregation and metabolic collapse. Cuproptosis can synergize with ferroptosis and immune activation, offering dual-mode lethality. Notably, FDX1, a key mediator of cuproptosis, correlates with immunosuppression and poor prognosis, suggesting its therapeutic relevance [188–192].

Apoptosis, as a classical programmed cell death pathway, remains a cornerstone in CNS tumor therapy. Targeted strategies, including BCL-2 inhibition, CD133 CRISPR editing, and mitochondrial dysfunction induction via agents such as quercetin, venetoclax, or Mito-LND, have shown efficacy in promoting apoptosis and sensitizing tumors to chemotherapy or radiotherapy. Inflammatory cytokine environments further sensitize glioma cells to apoptosis via TAK1 inhibition or caspase activation [193–199].

Autophagy, a double-edged sword in cancer, displays both tumor-promoting and tumor-suppressing roles. In gliomas, its activation may enhance therapy by promoting immunogenic HMGB1 release or reshaping TAMs toward a pro-inflammatory state. Agents like imipramine or alkylating drugs benefit from this mechanism. Conversely, autophagy inhibition, via agents disrupting autophagosome–lysosome fusion (e.g., regorafenib-Cu^2+^ systems and NEO214), can block survival pathways and overcome resistance, particularly in TMZ-refractory gliomas [200–204].

Overall, therapeutic exploitation of RCD pathways offers a multifaceted approach to CNS tumor treatment, particularly GBM, by directly inducing tumor cell death, reversing resistance phenotypes, and reshaping the immune microenvironment. Recent studies have demonstrated that the gut–brain axis microecology plays a pivotal role in regulating multiple forms of programmed cell death in CNS tumors, particularly ferroptosis and autophagy [107]. This regulation is mediated through microbial metabolites such as SCFAs, lactic acid, and LPSs, which influence the tumor immune microenvironment and therapeutic responsiveness. Moreover, dysbiosis of the intestinal microbiota can modulate glioma cell sensitivity to ferroptosis by altering inflammatory signaling and metabolic pathways, thereby offering novel therapeutic targets for CNS malignancies.

### Advanced drug delivery systems and platforms

The TME, which exhibits distinct characteristics including hypoxia, acidosis, and necrotic regions, creates favorable conditions for bacterial colonization, thereby enabling the development of bacteria-based platforms for targeted delivery of therapeutic agents [205,206]. Subsequently, engineered probiotics and bacteria-based tumor vaccines have evolved into a promising frontier in cancer therapeutics, particularly for CNS malignancies.

Emerging evidence indicates that bacteria-based therapeutic approaches provide distinct advantages, including precise controllability, enhanced immune activation, and potent oncolytic effects. Gurbatri et al. [206] engineered a probiotic-based delivery system for immune checkpoint inhibitors specific to CTLA-4 and PD-1, incorporating a proteolytic release mechanism to precisely control nanobody production and release. This system, in conjunction with probiotic-derived granulocyte-macrophage colony-stimulating factor (GM-CSF), effectively promoted T cell activation and enhanced immunological memory in tumor-bearing mice, resulting in robust systemic immune responses and tumor regression. The approach demonstrated superior safety and targeting efficiency compared to conventional delivery systems, optimizing checkpoint blockade therapy. Zhang et al. [207] developed a novel Salmonella delivery vector (SDV) utilizing attenuated *Salmonella typhimurium* (VNP20009) formulated within a hydrogel matrix. Following intracavitary administration, the system specifically targeted GBM cells, whereupon bacterial components triggered immune activation and enhanced phagocyte recruitment, facilitating tumor antigen presentation and inducing tumor cell pyroptosis. This therapeutic approach demonstrated significant efficacy against highly recurrent malignancies, particularly GBM multiforme. In advancing cancer vaccine strategies, Chen et al. [208] engineered an inactivated *E. coli* Nissle 1917 platform co-loaded with tumor antigens and β-glucan immunostimulant. Upon subcutaneous administration, the vaccine construct (BG/OVA@EcN) was efficiently internalized by macrophages, subsequently orchestrating DC recruitment and maturation, T cell activation, and M1-type macrophage polarization. This comprehensive immune response established trained immunity and immunological memory, conferring prophylactic protection, therapeutic efficacy, and recurrence prevention in tumor models.

### Therapeutic applications of nanomaterials

Nanomaterials, characterized by dimensions between 1 and 100 nm, offer distinctive advantages, including ultrafine particle size, superior drug loading capacity, enhanced physicochemical stability, and exceptional biocompatibility [209]. These unique properties have positioned nanomaterials as promising candidates for cancer therapeutics, particularly in addressing the challenges of brain tumor treatment.

**Fig. 9.**
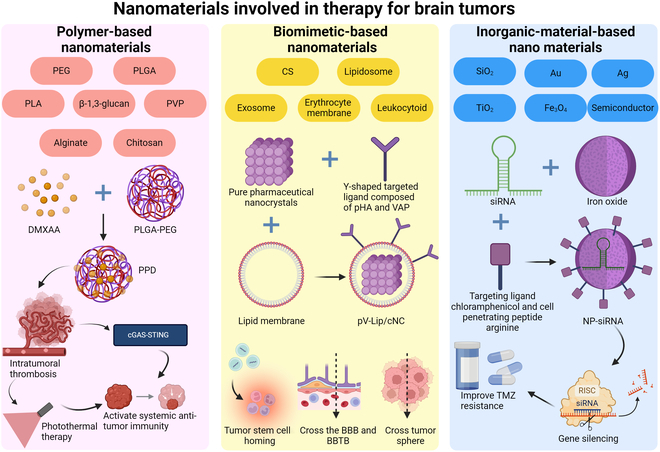
Nanomaterial-based strategies for brain tumor therapy.

Nanomaterials can be systematically categorized into 3 major classes: polymer-based, biomimetic, and inorganic platforms. Polymer-based platforms incorporate various biocompatible polymers, including polyethylene glycol (PEG), polylactic acid (PLA), poly (lactic-co-glycolic acid) (PLGA), β-1,3-d-glucan, polyvinylpyrrolidone (PVP), alginate, and chitosan (CS) [209]. In a pioneering research, Yin and colleagues engineered a novel PLGA-lysoGM1/DOX micellar system that effectively encapsulated doxorubicin (DOX), demonstrating controlled release kinetics and enhanced cellular internalization ex vivo. Subsequent in vivo experiments suggested efficient BBB penetration, selective tumor accumulation, and potent antitumor efficacy in GBM models [210]. Gu and colleagues [211] engineered a groundbreaking therapeutic delivery system by conjugating the vascular disrupting agent DMXAA with polyethylene glycol-grafted poly (lactic-co-glycolic acid) (PLGA-PEG) to synthesize PLGA-PEG/DMXAA (PPD) NPs. This innovative system facilitated photothermal therapy and simultaneously activated the cGAS–STING pathway, thereby promoting intratumoral thrombosis and eliciting systemic antitumor immune responses. Biomimetic nanocarriers encompass a diverse range of platforms, including CS, liposomes, exosomes, red blood cell membrane-derived vehicles, and biomimetic “leukocyte-like” particles [209]. Wu et al. [212] engineered a lipid membrane-coated cabazitaxel nanocrystal system (pV-Lip/cNC), wherein the lipid coating significantly enhanced stability and systemic circulation. Its core component is a drug nanocrystal, utilizing a pHA- and VAP-containing targeting ligand. When evaluated in glioma models, pV-Lip/cNC exhibited exceptional tumor-targeting capabilities, efficient penetration through both the BBB and blood–brain tumor barrier (BBTB), and superior tumor spheroid infiltration, culminating in robust tumor cell cytotoxicity. Wu and colleagues [213] developed a sophisticated delivery platform comprising AS1411 aptamer-modified macrophage exosomes containing catalase (CAT)-loaded silica NPs. This innovative platform exhibited enhanced BBB permeability, selective tumor cell targeting, exceptional biocompatibility, and extended circulation time in GBM treatment, highlighting its promise for clinical translation. Inorganic nanomaterials encompass diverse platforms, including silica-based structures, gold architectures, silver nanostructures, titanium dioxide constructs, iron oxide formulations, and semiconductor nanocrystals [209]. Zhang et al. [214], exploiting gold’s intrinsic properties and tumor-specific affinity, synthesized near-infrared-II (NIR-II)-responsive Au(I)-based ferroptosis nanoparticles using a thiolated benzothiazole-based phosphine (TBTP) ligand (TBTP-Au NPs), which demonstrated efficient BBB penetration, precise tumor targeting, and controlled ferroptosis induction. Chung et al. [215] engineered iron oxide NPs that generated stable siRNA complexes via electrostatic binding, facilitating efficient delivery to glioma cells. The incorporation of dual-targeting moieties—chlorotoxin and cell-penetrating peptide polyarginine—significantly enhanced siRNA transfection efficiency, achieving robust gene silencing and improved sensitization to TMZ therapy (Fig. 9).

Figure 9 illustrates the 3 main categories of nanomaterials utilized in brain tumor treatment: polymer-based nanomaterials (left), biomimetic nanomaterials (middle), and inorganic nanomaterials (right).

• Polymer-based nanomaterials: Examples include PEG, PLA, PLGA, β-1,3-d-glucan, PVP, alginate, and CS. For instance, Gu et al. developed PPD NPs by integrating the vascular-disrupting agent DMXAA into PLGA-PEG copolymers. These NPs mediate photothermal therapy and induce cGAS–STING pathway activation, triggering thrombosis within tumors and systemic antitumor immunity.
• Biomimetic nanomaterials: Common carriers include CS, liposomes, exosomes, red blood cell membranes, and leukocyte-mimicking systems. Wu et al. designed a pV-Lip/cNC. It comprises a lipid membrane-encapsulated core containing a pure drug nanocrystal, which is surface-functionalized with a Y-shaped pHA/VAP targeting ligand. It demonstrates exceptional capacities in crossing the BBB and BBTB, and penetrating tumor spheroids to effectively kill GBM cells.
• Inorganic nanomaterials: Examples include silica NPs, gold NPs, silver NPs, titanium dioxide NPs, iron oxide NPs, and semiconductor nanocrystals. For instance, Chung et al. developed iron oxide NPs that form complexes with siRNA through electrostatic interactions and successfully deliver the siRNA to GBM cells. Targeting ligands such as chlorotoxin and the cell-penetrating peptide polyarginine further enhance siRNA transfection efficiency, achieving high gene silencing rates and improved sensitivity to TMZ therapy.

Advances in nanotechnology have enabled the development of therapeutic strategies encompassing immune cell activation, targeted immune checkpoint blockade, and programmed cell death induction, substantially expanding the therapeutic arsenal for brain cancer treatment. Emerging evidence demonstrates that nanomaterial-based approaches significantly augment immune cell activation therapies. Zhu et al. engineered an innovative CAR-T cell platform (PLX-Lip/AZO-T cells) through the strategic conjugation of PLX-containing liposomes (PLX-Lip) with CAR-T cells. In a GBM mouse model, this system demonstrated efficient BBB penetration and preferential tumor accumulation. Following PLX-Lip uptake by TAMs in the TME, the platform induced M1-like macrophage polarization, thereby potentiating CAR-T cell-mediated tumor suppression. Zhang and colleagues [216] engineered a biomimetic NP system incorporating PLGA-coated TMZ and IL-15 NPs encased within cRGD-modified NK cell membranes (R-NKm@NP). Upon targeting the GBM site, R-NKm@NPs orchestrated comprehensive TME remodeling through controlled release of IL-15 and TMZ, which synergistically promoted NK cell multiplication and activation. This cascade further induced DC maturation and CD8^+^ cytotoxic T lymphocyte infiltration, resulting in robust anti-GBM efficacy. Extensive study underscores the promise of nanotechnology for therapeutic applications in enhancing immune checkpoint blockade therapies. Wang and colleagues [217] designed a BBB-permeable copolymer platform utilizing 2-methacryloyloxyethyl phosphorylcholine (MPC) via free radical polymerization, subsequently functionalizing it with anti-PD-L1 antibodies via pH-responsive cleavable linkers to generate therapeutic NPs. In preclinical GBM models, these NPs demonstrated efficient BBB penetration and facilitated selective anti-PD-L1 antibody release within the acidic TME, resulting in enhanced immune checkpoint blockade efficacy . Nie et al. [218] engineered a novel bioconjugate system by coupling azide-functionalized M1 macrophage-derived exosomes with dibenzocyclooctyne-modified anti-SIRPα and anti-CD47 antibodies via pH-responsive linkers . This engineered nano-bioconjugate system achieved tumor targeting through specific CD47 recognition and facilitated controlled release of anti-SIRPα and anti-CD47 antibodies within the acidic TME [218]. The dual-blocking mechanism effectively neutralized the “don’t eat me” signal by simultaneously targeting SIRPα on macrophages and CD47 on tumor cells, thereby circumventing tumor immune evasion[218]. Notably, nanotechnology-based approaches have demonstrated remarkable potential in modulating programmed cell death pathways. Wang et al. [219] engineered a sophisticated metal-organic complex comprising diethyldithiocarbamate (DDC) chelated with copper and ferrous ions (DDC/Cu-Fe), subsequently encapsulated within albumin and lactoferrin NPs (Alb/LF NP). This dual-targeted delivery system specifically recognized nutrient transporters in gliomas, facilitating enhanced DDC/Cu-Fe accumulation in the brain. The accumulated complexes effectively induced tumor cell ferroptosis, significantly extending survival in tumor-bearing mice. Xu et al. [220] developed copper selenide-coated gold NPs that systematically alkalized lysosomes, resulting in autophagic flux inhibition and suppression of protective autophagy in GBM. This mechanism concurrently enhanced GBM radiosensitivity through impairment of DNA repair pathways.

Currently, NP-based drug delivery technologies have entered the stage of clinical translation in certain brain tumors. Among them, liposome-based nanoparticles have been evaluated in multiple clinical trials for glioma treatment, demonstrating modest survival benefits. Notably, pegylated liposomes combined with chemotherapy have shown a median overall survival approximately 40 weeks longer than other second-line therapies. However, most clinical trials have failed to meet their primary efficacy endpoints and have not demonstrated significant clinical advantages. Nonetheless, with ongoing advancements and optimization in nanotechnology, the clinical application of NPs in brain tumor treatment continues to hold considerable promise [7].

## Discussion

CNS tumors are a major contributor to global cancer mortality, ranking second overall [221]. Contemporary standard-of-care treatments for CNS tumors primarily comprise surgical resection, multi-modal alkylating agent chemotherapy, and radiotherapy. The therapeutic efficacy is substantially compromised by the existence of the BBB and BBTB, coupled with the inherent heterogeneity of CNS tumors. These challenges contribute to high recurrence rates and the current absence of curative interventions [1]. Optimal therapeutic outcomes necessitate effective drug delivery and target specificity. Moreover, standard treatment-induced immunosuppression presents a significant barrier to immunotherapy implementation, constituting a critical challenge in the field [222]. This review comprehensively examines the gut–brain axis, encompassing CNS tumor classification, predictive models, and experimental approaches in gut microbiota research, including metabolomic profiles, microbiota modifications, and their mechanistic relationships with cell death pathways. Additionally, this review evaluates emerging immunotherapeutic and targeted therapeutic strategies for CNS tumors, with particular emphasis on the integration of advanced nanomaterial-based approaches.

This section elucidates the complex immune microenvironment of CNS cancers. The CNS TME is characterized by dysfunctional and depleted cytotoxic T cell populations, resulting from multifaceted suppression mediated by tumor cells, immunosuppressive factors, and diverse immune checkpoint pathways. Concurrently, tumor cells promote the enrichment of Tregs, while γδT cells, present in smaller populations, demonstrate a unique dual functionality in immunosurveillance and antitumor responses. TAMs, especially the immunosuppressive M2 phenotype, are regulated by complex mechanisms including endothelial cell (EC)-mediated sequential transcriptional activation via Twist1/SATB1 and the XCR2–JAK2/STAT3 signaling axis involving CXCL8. M2 TAMs up-regulate the CD47 “don’t eat me” signal, facilitating tumor immune evasion. MDSCs undergo expansion through CXCR-mediated recognition of tumor-derived CXCL1/2/3. Enhanced expression of tumor-associated adhesion molecules promotes MDSC recruitment, subsequently reinforcing the immunosuppressive microenvironment via the secretion of NO, TGF-β, and IL-10. NK cells exhibit dual functionality: Their antitumor activity is mediated by granzyme/perforin and Fas/FasL pathways while paradoxically promoting tumor malignant transformation via IFN-γ signaling. TANs demonstrate temporal phenotypic plasticity, comprising early-stage N1 phenotype, which induces tumor cell ferroptosis and necroptosis, and late-stage N2 phenotype, which facilitates tumor progression through the HMGB1/RAGE/IL-8 signaling axis. DCs demonstrate functional duality: They orchestrate antitumor responses through tumor antigen presentation while concurrently suppressing cytotoxic T cell recruitment and down-regulating costimulatory molecule expression, thereby promoting immunosuppression via IL-10 and TGF-β secretion.

Preclinical investigations of gut microbiota predominantly utilize murine and canine models. Prolonged antibiotic administration induces significant alterations in murine gut microbiota and associated metabolomic profiles in both gut and brain. These changes lead to reduced cytotoxic NK cell populations through gut–brain axis modulation, establishing a tumor-permissive CNS microenvironment and enhancing tumor growth through microglial and glioma cell-mediated angiogenesis. Canine studies implementing ketogenic dietary intervention demonstrated elevated levels of Fusobacteria and Bifidobacteria, with concurrent reduction in Lactobacilli populations in fecal microbiota. These alterations were accompanied by significant modifications in cholesterol/steroid metabolism and bile acid synthesis pathways, characterized by decreased valine, methionine, and Kyn levels, and elevated serotonin, suggesting enhanced resistance to glycolipid-dependent cancer stem cells. While clinical validation remains pending, extensive investigation is warranted to facilitate successful translational applications from preclinical findings to clinical practice.

With the rapid advancement of AI, radiomics, and molecular omics, various predictive models have demonstrated great potential in the diagnosis, treatment, and prognostic evaluation of CNS tumors. In particular, AI has enabled more accurate tumor classification and prediction of recurrence sites. Radiomics and imaging genomics, through the integration of imaging features and clinical data, can assist in risk stratification, assessment of tumor invasiveness, and prediction of immunotherapy response. Molecular omics-based models, such as those involving cuproptosis- or ferroptosis-related signatures, also offer promising tools for targeted drug screening and serve as potential prognostic biomarkers. However, most of these predictive models remain in the preclinical research phase. To facilitate clinical translation, it is essential to expand datasets, enhance external validation, and reduce dependence on methylation-based data, among other improvements.

Contemporary therapeutic approaches primarily encompass immune cell activation, immune checkpoint modulation, and programmed cell death induction. Selective activation of T lymphocytes, neutrophils, NK cells, and macrophages in the CNS TME facilitates the reversal of immunosuppression and enhances antitumor responses through dual mechanisms: immunosuppression antagonism and enhanced phagocytic activity. Therapeutic antibodies and checkpoint inhibitors targeting diverse immune checkpoint pathways effectively alleviate tumor-mediated immune cell suppression, thereby reinvigorating antitumor immunity. Engagement of multiple cell death pathways, including ferroptosis, copper-dependent cell death, apoptosis, and autophagy, contributes to significant tumor growth inhibition and enhanced therapeutic responsiveness. Integration of these targeted approaches with advanced engineered probiotics, cancer vaccines, and nanomaterial-based delivery platforms presents compelling translational potential for clinical applications. This comprehensive therapeutic strategy is anticipated to enhance treatment outcomes through multiple mechanisms: overcoming resistance to standard therapies, facilitating drug penetration across the BBB and BTB, enabling precise tumor targeting, expanding available treatment options, and ultimately improving therapeutic efficacy in brain cancer management.

Several limitations warrant consideration in this review, particularly regarding the depth of mechanistic analyses. Current investigations of the gut–brain axis predominantly rely on murine and canine models, which inadequately represent the intricate pathological crosstalk between human gut microbiota and brain tumor pathogenesis. When using animal models to investigate the relationship between gut microbiota and brain tumors, several key limitations arise due to interspecies physiological and microbiological differences. These include disparities in intestinal structure and microbiota composition, BBB permeability, immune system responses, and the mechanisms underlying brain tumor initiation and progression. Moreover, the standardized diets and specific pathogen-free (SPF) environments in which laboratory animals are maintained differ markedly from real-life human conditions, further limiting the clinical translatability of findings derived from such models. Notably, emerging 3D bioprinted gut models offer a promising alternative, as they can more accurately replicate the complex architecture and hypoxic microenvironment of the human intestine. These models hold significant potential for advancing research on the gut–brain axis and for developing in vitro platforms to explore microbiota-based interventions for brain tumors. As such, they may represent a critical direction for future research [81]. The absence of large-scale cohort studies impedes robust statistical validation of the proposed associations between gut–brain axis dysregulation and brain tumorigenesis. The inherent complexity of gut–brain axis research, encompassing interactions among the CNS, digestive system, and immune system, necessitates further comprehensive investigation to elucidate underlying mechanisms.

Emerging immunotherapeutic and targeted approaches, including CAR-T cell therapy, PD-1 pathway blockade, CD47-targeted nanotherapeutics, engineered probiotics, and ferroptosis-inducing cancer vaccines, demonstrate substantial promise in brain tumor treatment. Notably, recent findings suggest that the gut–brain axis and microbiota-derived metabolites, such as SCFAs, tryptophan catabolites, and secondary bile acids, play critical immunomodulatory roles that may synergize with these therapies. For instance, SCFAs regulate γδ T cell activity and IL-17 production to adjust the TME responsiveness to checkpoint blockade. Additionally, engineered probiotics offer a novel route for local or systemic delivery of immune-activating molecules, further linking microbiota dynamics to antitumor immunity. However, the underlying mechanisms of host–microbiota interactions in CNS tumors remain incompletely understood. Interindividual variability in microbiome composition, BBB integrity, and metabolite availability may all contribute to inconsistent therapeutic responses. Furthermore, the safety and efficacy of gut-targeted interventions in glioma patients, who often have disrupted gut integrity due to chemotherapy or corticosteroids, require cautious validation.

Probiotic interventions, particularly those involving engineered strains, represent promising avenues for delivering immunomodulating agents in a targeted manner, either locally or systemically, and may be tailored to reshape the tumor immune microenvironment. Fecal microbiota transplantation (FMT) has also shown potential in restoring microbial diversity and enhancing antitumor immunity in other cancer contexts. Nevertheless, its application in CNS tumors must be carefully evaluated, especially considering the altered gastrointestinal environment often seen in glioma patients undergoing treatment. Furthermore, targeted modulation of microbial metabolites, such as SCFA supplementation or manipulation of the tryptophan metabolic pathway, holds potential to reshape both systemic and CNS-specific immune responses [223]. Microbiome engineering, as a next-generation strategy, enables precise modulation of host–microbiota interactions, offering opportunities to enhance therapeutic efficacy while minimizing off-target effects. Taken together, these strategies present exciting prospects for clinical translation. Nonetheless, challenges remain, particularly those related to interindividual variability in microbiome profiles, BBB permeability, and metabolite bioavailability. Therefore, further mechanistic investigations and well-controlled clinical trials are urgently needed to determine therapeutic efficacy, optimize patient selection, and ensure safety in CNS tumor populations. Highlighting these approaches will help establish a more comprehensive roadmap for the integration of microbiota-targeted therapies into brain tumor treatment paradigms.

Currently, there is no direct clinical evidence indicating that individuals with gut microbiota dysbiosis are more susceptible to CNS tumors. However, several preclinical studies and clinical observations in brain tumor patients suggest a potential association between gut dysbiosis and CNS tumorigenesis. For example, fecal DNA sequencing analyses in patients with malignant brain tumors have revealed a significant reduction in microbial diversity, accompanied by an increased relative abundance of Bacteroides, Fusobacteria, and Proteobacteria, and a decreased abundance of Firmicutes and Actinomycetes. In addition, animal studies have shown pronounced disruptions in the gut microbiota of mice implanted with gliomas, further supporting the potential involvement of the gut–brain–microbiota axis in brain tumor development [83]. A recent Mendelian randomization study provided further insight by identifying a positive correlation between brain cancer risk and the presence of Lactobacillus and Clostridium family 1, and a negative correlation with Defluviitaleaceae UCG-011 and Flavonifractor. This was the first study to suggest a potential causal link between gut microbial imbalance and the development of brain cancer [224]. Building on these findings, an increasing number of studies are now exploring the integration of immunotherapy with microbiome modulation to enhance therapeutic efficacy against brain tumors.

Future research should aim to delineate the causal relationships between specific microbial signatures and treatment outcomes, identify reliable microbiome-derived biomarkers for patient stratification, and develop rational combination strategies involving microbiome modulation and CNS-targeted immunotherapies. Ultimately, leveraging the gut–brain–immune axis may unlock personalized, microbiota-informed approaches to enhance treatment efficacy and improve prognosis in brain tumor patients.

## Data Availability

This review is based on previously published studies. No new data were generated or analyzed in this study. All referenced data can be accessed through the cited publications.

## References

[1] Schaff LR, Mellinghoff IK. Glioblastoma and other primary brain malignancies in adults: A review. JAMA. 2023;329(7):574–587. 36809318 10.1001/jama.2023.0023 PMC11445779

[2] Yang K, Wu Z, Zhang H, Zhang N, Wu W, Wang Z, Dai Z, Zhang X, Zhang L, Peng Y, et al. Glioma targeted therapy: Insight into future of molecular approaches. Mol Cancer. 2022;21(1):39. 35135556 10.1186/s12943-022-01513-z PMC8822752

[3] Weller M, Wen PY, Chang SM, Dirven L, Lim M, Monje M, Reifenberger G. Glioma. Nat Rev Dis Primers. 2024;10(1):33. 38724526 10.1038/s41572-024-00516-y

[4] Gritsch S, Batchelor TT, Gonzalez Castro LN. Diagnostic, therapeutic, and prognostic implications of the 2021 World Health Organization classification of tumors of the central nervous system. Cancer. 2022;128(1):47–58. 34633681 10.1002/cncr.33918

[5] Sinicrope K, Batchelor T. Primary central nervous system lymphoma. Neurol Clin. 2018;36(3):517–532. 30072069 10.1016/j.ncl.2018.04.008

[6] Träger M, Schweizer L, Pérez E, Schmid S, Hain EG, Dittmayer C, Onken J, Fukuoka K, Ichimura K, Schüller U, et al. Adult intracranial ependymoma-relevance of DNA methylation profiling for diagnosis, prognosis, and treatment. Neuro-Oncology. 2023;25(7):1286–1298. 36734226 10.1093/neuonc/noad030 PMC10326475

[7] Lai G, Wu H, Yang K, Hu K, Zhou Y, Chen X, Fu F, Li J, Xie G, Wang HF, et al. Progress of nanoparticle drug delivery system for the treatment of glioma. Front Bioeng Biotechnol. 2024;12:1403511. 38919382 10.3389/fbioe.2024.1403511 PMC11196769

[8] Ruda R, Bruno F, Pellerino A, Soffietti R. Ependymoma: Evaluation and management updates. Curr Oncol Rep. 2022;24(8):985–993. 35384591 10.1007/s11912-022-01260-w PMC9249684

[9] Vainchtein ID, Molofsky AV. Astrocytes and microglia: In sickness and in health. Trends Neurosci. 2020;43(3):144–154. 32044129 10.1016/j.tins.2020.01.003 PMC7472912

[10] Langen UH, Ayloo S, Gu C. Development and cell biology of the blood-brain barrier. Annu Rev Cell Dev Biol. 2019;35(1):591–613. 31299172 10.1146/annurev-cellbio-100617-062608 PMC8934576

[11] Smith BC, Tinkey RA, Shaw BC, Williams JL. Targetability of the neurovascular unit in inflammatory diseases of the central nervous system. Immunol Rev. 2022;311(1):39–49. 35909222 10.1111/imr.13121 PMC9489669

[12] Kipp M. Oligodendrocyte physiology and pathology function. Cells. 2020;9(9):2078. 32932835 10.3390/cells9092078 PMC7563511

[13] Croese T, Castellani G, Schwartz M. Immune cell compartmentalization for brain surveillance and protection. Nat Immunol. 2021;22(9):1083–1092. 34429552 10.1038/s41590-021-00994-2

[14] Prinz M, Masuda T, Wheeler MA, Quintana FJ. Microglia and central nervous system-associated macrophages—From origin to disease modulation. Annu Rev Immunol. 2021;39:251–277. 33556248 10.1146/annurev-immunol-093019-110159 PMC8085109

[15] Sun R, Jiang H. Border-associated macrophages in the central nervous system. J Neuroinflammation. 2024;21(1):67. 38481312 10.1186/s12974-024-03059-x PMC10938757

[16] Muzio L, Perego J. CNS resident innate immune cells: Guardians of CNS homeostasis. Int J Mol Sci. 2024;25(9):4865. 38732082 10.3390/ijms25094865 PMC11084235

[17] Wang Y, Sadike D, Huang B, Li P, Wu Q, Jiang N, Fang Y, Song G, Xu L, Wang W, et al. Regulatory T cells alleviate myelin loss and cognitive dysfunction by regulating neuroinflammation and microglial pyroptosis via TLR4/MyD88/NF-kappaB pathway in LPC-induced demyelination. J Neuroinflammation. 2023;20(1):41. 36803990 10.1186/s12974-023-02721-0 PMC9938996

[18] Sabatino JJ Jr, Probstel AK, Zamvil SS. B cells in autoimmune and neurodegenerative central nervous system diseases. Nat Rev Neurosci. 2019;20(12):728–745. 31712781 10.1038/s41583-019-0233-2

[19] Manda-Handzlik A, Demkow U. The brain entangled: The contribution of neutrophil extracellular traps to the diseases of the central nervous system. Cells. 2019;8(12):1477. 31766346 10.3390/cells8121477 PMC6953104

[20] Sandhu JK, Kulka M. Decoding mast cell-microglia communication in neurodegenerative diseases. Int J Mol Sci. 2021;22(3):1093. 33499208 10.3390/ijms22031093 PMC7865982

[21] Balatsoukas A, Rossignoli F, Shah K. NK cells in the brain: Implications for brain tumor development and therapy. Trends Mol Med. 2022;28(3):194–209. 35078713 10.1016/j.molmed.2021.12.008 PMC8882142

[22] Wyatt-Johnson SK, Afify R, Brutkiewicz RR. The immune system in neurological diseases: What innate-like T cells have to say. J Allergy Clin Immunol. 2024;153(4):913–923. 38365015 10.1016/j.jaci.2024.02.003 PMC10999338

[23] Wo J, Zhang F, Li Z, Sun C, Zhang W, Sun G. The role of gamma-delta T cells in diseases of the central nervous system. Front Immunol. 2020;11: Article 580304. 33193380 10.3389/fimmu.2020.580304 PMC7644879

[24] Becher B, Spath S, Goverman J. Cytokine networks in neuroinflammation. Nat Rev Immunol. 2017;17(1):49–59. 27916979 10.1038/nri.2016.123

[25] Gonzalez Caldito N. Role of tumor necrosis factor-alpha in the central nervous system: A focus on autoimmune disorders. Front Immunol. 2023;14:1213448. 37483590 10.3389/fimmu.2023.1213448 PMC10360935

[26] Kalluri AL, Shah PP, Lim M. The tumor immune microenvironment in primary CNS neoplasms: A review of current knowledge and therapeutic approaches. Int J Mol Sci. 2023;24(3):2020. 36768342 10.3390/ijms24032020 PMC9917056

[27] Qi Y, Hu L, Ji C, Yang X, Yao J, Chen D, Yao Y. B7-H4 reduces the infiltration of CD^8+^T cells and induces their anti-tumor dysfunction in gliomas. Neoplasia. 2024;54: Article 101007. 38796932 10.1016/j.neo.2024.101007 PMC11152750

[28] Bikfalvi A, da Costa CA, Avril T, Barnier JV, Bauchet L, Brisson L, Cartron PF, Castel H, Chevet E, Chneiweiss H, et al. Challenges in glioblastoma research: Focus on the tumor microenvironment. Trends Cancer. 2023;9(1):9–27. 36400694 10.1016/j.trecan.2022.09.005

[29] Gieryng A, Pszczolkowska D, Walentynowicz KA, Rajan WD, Kaminska B. Immune microenvironment of gliomas. Lab Investig. 2017;97(5):498–518. 28287634 10.1038/labinvest.2017.19

[30] Li W, Zhao X, Ren C, Gao S, Han Q, Lu M, Li X. The therapeutic role of γδT cells in TNBC. Front Immunol. 2024;15:1420107. 38933280 10.3389/fimmu.2024.1420107 PMC11199784

[31] Choi H, Kim TG, Jeun SS, Ahn S. Human gamma-delta (γδ) T cell therapy for glioblastoma: A novel alternative to overcome challenges of adoptive immune cell therapy. Cancer Lett. 2023;571: Article 216335. 37544475 10.1016/j.canlet.2023.216335

[32] Choi H, Lee Y, Park SA, Lee JH, Park J, Park JH, Lee HK, Kim TG, Jeun SS, Ahn S. Human allogenic γδ T cells kill patient-derived glioblastoma cells expressing high levels of DNAM-1 ligands. Onco Targets Ther. 2022;11(1):2138152. 10.1080/2162402X.2022.2138152 PMC9629076 36338147

[33] Yan J. Antitumor γδ T cells need oxygen to function. Nat Immunol. 2021;22(3):268–269. 33574620 10.1038/s41590-021-00874-9 PMC11418018

[34] Guadagno E, Presta I, Maisano D, Donato A, Pirrone C, Cardillo G, Corrado S, Mignogna C, Mancuso T, Donato G, et al. Role of macrophages in brain tumor growth and progression. Int J Mol Sci. 2018;19(4):1005. 29584702 10.3390/ijms19041005 PMC5979398

[35] Yang F, Akhtar MN, Zhang D, el-Mayta R, Shin J, Dorsey JF, Zhang L, Xu X, Guo W, Bagley SJ, et al. An immunosuppressive vascular niche drives macrophage polarization and immunotherapy resistance in glioblastoma. Sci Adv. 2024;10(9): Article eadj4678. 38416830 10.1126/sciadv.adj4678 PMC10901371

[36] Guo X, Qiu W, Li B, Qi Y, Wang S, Zhao R, Cheng B, Han X, du H, Pan Z, et al. Hypoxia-induced neuronal activity in glioma patients polarizes microglia by potentiating RNA m6A demethylation. Clin Cancer Res. 2024;30(6):1160–1174. 37855702 10.1158/1078-0432.CCR-23-0430

[37] Song Y, Zhang Y, Wang Z, Lin Y, Cao X, Han X, Li G, Hou A, Han S. CCL2 mediated IKZF1 expression promotes M2 polarization of glioma-associated macrophages through CD84-SHP2 pathway. Oncogene. 2024;43(36):2737–2749. 39112517 10.1038/s41388-024-03118-w

[38] Yuan W, Zhang Q, Gu D, Lu C, Dixit D, Gimple RC, Gao Y, Gao J, Li D, Shan D, et al. Dual role of CXCL8 in maintaining the mesenchymal state of glioblastoma stem cells and M2-like tumor-associated macrophages. Clin Cancer Res. 2023;29(18):3779–3792. 37439870 10.1158/1078-0432.CCR-22-3273

[39] Yan C, Yang Z, Chen P, Yeh Y, Sun C, Xie T, Huang W, Zhang X. GPR65 sensing tumor-derived lactate induces HMGB1 release from TAM via the cAMP/PKA/CREB pathway to promote glioma progression. J Exp Clin Cancer Res. 2024;43(1):105. 38576043 10.1186/s13046-024-03025-8 PMC10993467

[40] Liu X, Zhang H, Wang C, Li Z, Zhu Q, Feng Y, Fan J, Qi S, Wu Z, Liu Y. Tumor-selective blockade of CD47 signaling with CD47 antibody for enhanced anti-tumor activity in malignant meningioma. *CNS Neurol Disord Drug Targets*. 2023;22(8):e230511123157. 10.2174/1570159X21666230511123157 PMC10556363 37171006

[41] Song D, Yang Q, Li L, Wei Y, Zhang C, du H, Ren G, Li H. Novel prognostic biomarker TBC1D1 is associated with immunotherapy resistance in gliomas. Front Immunol. 2024;15:1372113. 38529286 10.3389/fimmu.2024.1372113 PMC10961388

[42] Yang M, Wang B, Yin Y, Ma X, Tang L, Zhang Y, Fan Q, Yin T, Wang Y. PTN-PTPRZ1 signaling axis blocking mediates tumor microenvironment remodeling for enhanced glioblastoma treatment. J Control Release. 2023;353:63–76. 36402232 10.1016/j.jconrel.2022.11.025

[43] Kloosterman DJ, Erbani J, Boon M, Farber M, Handgraaf SM, Ando-Kuri M, Sánchez-López E, Fontein B, Mertz M, Nieuwland M, et al. Macrophage-mediated myelin recycling fuels brain cancer malignancy. Cell. 2024;187(19):5336–5356.e30. 39137777 10.1016/j.cell.2024.07.030 PMC11429458

[44] Wang W, Li T, Cheng Y, Li F, Qi S, Mao M, Wu J, Liu Q, Zhang X, Li X, et al. Identification of hypoxic macrophages in glioblastoma with therapeutic potential for vasculature normalization. Cancer Cell. 2024;42(5):815–832.e12. 38640932 10.1016/j.ccell.2024.03.013

[45] Yeo AT, Shah R, Aliazis K, Pal R, Xu T, Zhang P, Rawal S, Rose CM, Varn FS, Appleman VA, et al. Driver mutations dictate the immunologic landscape and response to checkpoint immunotherapy of glioblastoma. Cancer Immunol Res. 2023;11(5):629–645. 36881002 10.1158/2326-6066.CIR-22-0655 PMC10155040

[46] Yu T, Wang K, Wang J, Liu Y, Meng T, Hu F, Yuan H. M-MDSCs mediated trans-BBB drug delivery for suppression of glioblastoma recurrence post-standard treatment. J Control Release. 2024;369:199–214. 38537717 10.1016/j.jconrel.2024.03.043

[47] Zhou Y, Cheng L, Liu L, Li X. NK cells are never alone: Crosstalk and communication in tumour microenvironments. Mol Cancer. 2023;22(1):34. 36797782 10.1186/s12943-023-01737-7 PMC9933398

[48] Sun C, Wang S, Ma Z, Zhou J, Ding Z, Yuan G, Pan Y. Neutrophils in glioma microenvironment: From immune function to immunotherapy. Front Immunol. 2024;15:1393173. 38779679 10.3389/fimmu.2024.1393173 PMC11109384

[49] Tsai HC, Tong ZJ, Hwang TL, Wei KC, Chen PY, Huang CY, Chen KT, Lin YJ, Cheng HW, Wang HT. Acrolein produced by glioma cells under hypoxia inhibits neutrophil Akt activity and suppresses anti-tumoral activities. Free Radic Biol Med. 2023;207:17–28. 37414347 10.1016/j.freeradbiomed.2023.06.027

[50] Zha C, Meng X, Li L, Mi S, Qian D, Li Z, Wu P, Hu S, Zhao S, Cai J, et al. Neutrophil extracellular traps mediate the crosstalk between glioma progression and the tumor microenvironment via the HMGB1/RAGE/IL-8 axis. Cancer Biol Med. 2020;17(1):154–168. 32296583 10.20892/j.issn.2095-3941.2019.0353 PMC7142852

[51] Lu T, Yee PP, Chih SY, Tang M, Chen H, Aregawi DG, Glantz MJ, Zacharia BE, Wang HG, Li W. LC3-associated phagocytosis of neutrophils triggers tumor ferroptotic cell death in glioblastoma. EMBO J. 2024;43(13):2582–2605. 38806658 10.1038/s44318-024-00130-4 PMC11217441

[52] Cheng S, Liu L, Wang D, Li Y, Li S, Yuan J, Huang S, Xu Z, Jia B, Li Z, et al. Upregulation of the ZNF148/PTX3 axis promotes malignant transformation of dendritic cells in glioma stem-like cells microenvironment. CNS Neurosci Ther. 2023;29(9):2690–2704. 37063077 10.1111/cns.14213 PMC10401131

[53] Yang J, Zhang M, Zhang X, Zhou Y, Ma T, Liang J, Zhang J. Glioblastoma-derived exosomes promote lipid accumulation and induce ferroptosis in dendritic cells via the NRF2/GPX4 pathway. Front Immunol. 2024;15:1439191. 39192971 10.3389/fimmu.2024.1439191 PMC11347305

[54] Hoang DT, Shulman ED, Turakulov R, Abdullaev Z, Singh O, Campagnolo EM, Lalchungnunga H, Stone EA, Nasrallah MLP, Ruppin E, et al. Prediction of DNA methylation-based tumor types from histopathology in central nervous system tumors with deep learning. Nat Med. 2024;30(7):1952–1961. 38760587 10.1038/s41591-024-02995-8

[55] Joo L, Park JE, Park SY, Nam SJ, Kim YH, Kim JH, Kim HS. Extensive peritumoral edema and brain-to-tumor interface MRI features enable prediction of brain invasion in meningioma: Development and validation. Neuro-Oncology. 2021;23(2):324–333. 32789495 10.1093/neuonc/noaa190 PMC8631067

[56] Chen B, Zhou X, Yang L, Zhou H, Meng M, Zhang L, Li J. A Cuproptosis activation scoring model predicts neoplasm-immunity interactions and personalized treatments in glioma. Comput Biol Med. 2022;148: Article 105924. 35964468 10.1016/j.compbiomed.2022.105924

[57] Han L, Zhou J, Li L, Wu X, Shi Y, Cui W, Zhang S, Hu Q, Wang J, Bai H, et al. SLC1A5 enhances malignant phenotypes through modulating ferroptosis status and immune microenvironment in glioma. Cell Death Dis. 2022;13(12):1071. 36566214 10.1038/s41419-022-05526-w PMC9789994

[58] Nam SJ, Kim YH, Park JE, Ra YS, Khang SK, Cho YH, Kim JH, Sung CO. Tumor-infiltrating immune cell subpopulations and programmed death ligand 1 (PD-L1) expression associated with clinicopathological and prognostic parameters in ependymoma. Cancer Immunol Immunother. 2019;68(2):305–318. 30483834 10.1007/s00262-018-2278-x PMC11028367

[59] Heger JM, Mattlener J, Schneider J, Gödel P, Sieg N, Ullrich F, Lewis R, Bucaciuc-Mracica T, Schwarz RF, Rueß D, et al. Entirely noninvasive outcome prediction in central nervous system lymphomas using circulating tumor DNA. Blood. 2024;143(6):522–534. 37946299 10.1182/blood.2023022020

[60] Herrgott GA, Snyder JM, She R, Malta TM, Sabedot TS, Lee IY, Pawloski J, Podolsky-Gondim GG, Asmaro KP, Zhang J, et al. Detection of diagnostic and prognostic methylation-based signatures in liquid biopsy specimens from patients with meningiomas. Nat Commun. 2023;14(1):5669. 37704607 10.1038/s41467-023-41434-z PMC10499807

[61] Zhou T, Noeuveglise A, Modzelewski R, Ghazouani F, Thureau S, Fontanilles M, Ruan S. Prediction of brain tumor recurrence location based on multi-modal fusion and nonlinear correlation learning. Comput Med Imaging Graph. 2023;106: Article 102218. 36947921 10.1016/j.compmedimag.2023.102218

[62] Fan X, Li J, Huang B, Lu H, Lu C, Pan M, Wang X, Zhang H, You Y, Wang X, et al. Noninvasive radiomics model reveals macrophage infiltration in glioma. Cancer Lett. 2023;573: Article 216380. 37660885 10.1016/j.canlet.2023.216380

[63] Losno EA, Sieferle K, Perez-Cueto FJA, Ritz C. Vegan diet and the gut microbiota composition in healthy adults. Nutrients. 2021;13(7):2402. 34371912 10.3390/nu13072402 PMC8308632

[64] Hsu CL, Schnabl B. The gut-liver axis and gut microbiota in health and liver disease. Nat Rev Microbiol. 2023;21(11):719–733. 37316582 10.1038/s41579-023-00904-3 PMC10794111

[65] Tewari N, Dey P. Navigating commensal dysbiosis: Gastrointestinal host-pathogen interplay orchestrating opportunistic infections. Microbiol Res. 2024;286: Article 127832. 39013300 10.1016/j.micres.2024.127832

[66] Song Z, Song R, Liu Y, Wu Z, Zhang X. Effects of ultra-processed foods on the microbiota-gut-brain axis: The bread-and-butter issue. Food Res Int. 2023;167: Article 112730. 37087282 10.1016/j.foodres.2023.112730

[67] Liu Y, Jiao C, Zhang T, Li X, Li P, Lu M, Ye Z, du Y, du R, Zhang W, et al. Early-life gut microbiota governs susceptibility to colitis via microbial-derived ether lipids. Research. 2023;6:0037. 37040489 10.34133/research.0037 PMC10076029

[68] Tie Y, Huang Y, Chen R, Li L, Chen M, Zhang S. Current insights on the roles of gut microbiota in inflammatory bowel disease-associated extra-intestinal manifestations: Pathophysiology and therapeutic targets. Gut Microbes. 2023;15(2):2265028. 37822139 10.1080/19490976.2023.2265028 PMC10572083

[69] Martin CR, Osadchiy V, Kalani A, Mayer EA. The brain-gut-microbiome axis. Cell Mol Gastroenterol Hepatol. 2018;6(2):133–148. 30023410 10.1016/j.jcmgh.2018.04.003 PMC6047317

[70] Cryan JF, O’Riordan KJ, Sandhu K, Peterson V, Dinan TG. The gut microbiome in neurological disorders. Lancet Neurol. 2020;19(2):179–194. 31753762 10.1016/S1474-4422(19)30356-4

[71] Cryan JF, O’Riordan KJ, Cowan CSM, Sandhu KV, Bastiaanssen TFS, Boehme M, Codagnone MG, Cussotto S, Fulling C, Golubeva AV, et al. The microbiota-gut-brain axis. Physiol Rev. 2019;99(4):1877–2013. 31460832 10.1152/physrev.00018.2018

[72] Lin X, Yu Z, Liu Y, Li C, Hu H, Hu JC, Liu M, Yang Q, Gu P, Li J. Gut–X axis. iMeta. 2025;4(1): Article e270. 40027477 10.1002/imt2.270 PMC11865426

[73] Jin K, Chen B, Han S, Dong J, Cheng S, Qin B, Lu J. Repetitive transcranial magnetic stimulation (rTMS) improves cognitive impairment and intestinal microecological dysfunction induced by high-fat diet in rats. Research. 2024;7:0384. 38826566 10.34133/research.0384 PMC11140411

[74] Gao B, Chi L, Zhu Y, Shi X, Tu P, Li B, Yin J, Gao N, Shen W, Schnabl B. An introduction to next generation sequencing bioinformatic analysis in gut microbiome studies. Biomolecules. 2021;11(4):530. 33918473 10.3390/biom11040530 PMC8066849

[75] Wensel CR, Pluznick JL, Salzberg SL, Sears CL. Next-generation sequencing: Insights to advance clinical investigations of the microbiome. J Clin Invest. 2022;132(7): Article e154944. 35362479 10.1172/JCI154944 PMC8970668

[76] Morais LH, Schreiber HLT, Mazmanian SK. The gut microbiota-brain axis in behaviour and brain disorders. Nat Rev Microbiol. 2021;19(4):241–255. 33093662 10.1038/s41579-020-00460-0

[77] Fröhlich EE, Farzi A, Mayerhofer R, Reichmann F, Jačan A, Wagner B, Zinser E, Bordag N, Magnes C, Fröhlich E, et al. Cognitive impairment by antibiotic-induced gut dysbiosis: Analysis of gut microbiota-brain communication. Brain Behav Immun. 2016;56:140–155. 26923630 10.1016/j.bbi.2016.02.020 PMC5014122

[78] D’Alessandro G, Antonangeli F, Marrocco F, Porzia A, Lauro C, Santoni A, Limatola C. Gut microbiota alterations affect glioma growth and innate immune cells involved in tumor immunosurveillance in mice. Eur J Immunol. 2020;50(5):705–711. 32034922 10.1002/eji.201948354 PMC7216943

[79] Rosito M, Maqbool J, Reccagni A, Giampaoli O, Sciubba F, Antonangeli F, Scavizzi F, Raspa M, Cordella F, Tondo L, et al. Antibiotics treatment promotes vasculogenesis in the brain of glioma-bearing mice. Cell Death Dis. 2024;15(3):210. 38480690 10.1038/s41419-024-06578-w PMC10937980

[80] Allenspach K, Borcherding DC, Iennarella-Servantez CA, Mosichuk AP, Atherly T, Sahoo DK, Kathrani A, Suchodolski JS, Bourgois-Mochel A, Serao MR, et al. Ketogenic diets in healthy dogs induce gut and serum metabolome changes suggestive of anti-tumourigenic effects: A model for human ketotherapy trials. Clin Transl Med. 2022;12(9): Article e1047. 10.1002/ctm2.1047 PMC9506423 36149786

[81] Cheng L, Liu T, Liu Q, Lian L, Tang G, Mille LS, García FR, Engstrand L, Zhang YS, du J. A 3D bioprinted gut anaerobic model for studying bacteria-host interactions. Research. 2023;6:0058. 37040488 10.34133/research.0058 PMC10076011

[82] Yang Q, Wang B, Zheng Q, Li H, Meng X, Zhou F, Zhang L. A review of gut microbiota-derived metabolites in tumor progression and cancer therapy. Adv Sci. 2023;10(15): Article e2207366. 10.1002/advs.202207366 PMC10214247 36951547

[83] Aljarrah D, Chalour N, Zorgani A, Nissan T, Pranjol MZI. Exploring the gut microbiota and its potential as a biomarker in gliomas. Biomed Pharmacother. 2024;173: Article 116420. 38471271 10.1016/j.biopha.2024.116420

[84] Wang J, Zhu N, Su X, Gao Y, Yang R. Gut-microbiota-derived metabolites maintain gut and systemic immune homeostasis. Cells. 2023;12(5):793. 36899929 10.3390/cells12050793 PMC10000530

[85] Sadik A, Somarribas Patterson LF, Öztürk S, Mohapatra SR, Panitz V, Secker PF, Pfänder P, Loth S, Salem H, Prentzell MT, et al. IL4I1 is a metabolic immune checkpoint that activates the AHR and promotes tumor progression. Cell. 2020;182(5):1252–1270.e34. 32818467 10.1016/j.cell.2020.07.038

[86] Lyu Y, Yang H, Chen L. Metabolic regulation on the immune environment of glioma through gut microbiota. Semin Cancer Biol. 2022;86(Pt 2):990–997. 33971263 10.1016/j.semcancer.2021.05.005

[87] Wang W, Ou Z, Huang X, Wang J, Li Q, Wen M, Zheng L. Microbiota and glioma: A new perspective from association to clinical translation. Gut Microbes. 2024;16(1):2394166. 39185670 10.1080/19490976.2024.2394166 PMC11352717

[88] He Y, Fu L, Li Y, Wang W, Gong M, Zhang J, Dong X, Huang J, Wang Q, Mackay CR, et al. Gut microbial metabolites facilitate anticancer therapy efficacy by modulating cytotoxic CD8^+^ T cell immunity. Cell Metab. 2021;33(5):988–1000.e7. 33761313 10.1016/j.cmet.2021.03.002

[89] Mohamed AA, Al-Ramadi BK, Fernandez-Cabezudo MJ. Interplay between microbiota and γδ T cells: Insights into immune homeostasis and neuro-immune interactions. Int J Mol Sci. 2024;25(3):1747. 38339023 10.3390/ijms25031747 PMC10855551

[90] Dehhaghi M, Kazemi Shariat Panahi H, Heng B, Guillemin GJ. The gut microbiota, kynurenine pathway, and immune system interaction in the development of brain cancer. Front Cell Dev Biol. 2020;8: Article 562812. 33330446 10.3389/fcell.2020.562812 PMC7710763

[91] Riess C, del Moral K, Fiebig A, Kaps P, Linke C, Hinz B, Rupprecht A, Frank M, Fiedler T, Koczan D, et al. Implementation of a combined CDK inhibition and arginine-deprivation approach to target arginine-auxotrophic glioblastoma multiforme cells. Cell Death Dis. 2022;13(6):555. 35717443 10.1038/s41419-022-05006-1 PMC9206658

[92] Yuxiao C, Jiachen W, Yanjie L, Shenglan L, Yuji W, Wenbin L. Therapeutic potential of arginine deprivation therapy for gliomas: A systematic review of the existing literature. Front Pharmacol. 2024;15:1446725. 39239650 10.3389/fphar.2024.1446725 PMC11375294

[93] Zou Z, Cheng Q, Zhou J, Guo C, Hadjinicolaou AV, Salio M, Liang X, Yang C, du Y, Yao W, et al. ATF4-SLC7A11-GSH axis mediates the acquisition of immunosuppressive properties by activated CD4^+^ T cells in low arginine condition. Cell Rep. 2024;43(4): Article 113995. 38527061 10.1016/j.celrep.2024.113995

[94] Platten M, Friedrich M, Wainwright DA, Panitz V, Opitz CA. Tryptophan metabolism in brain tumors—IDO and beyond. Curr Opin Immunol. 2021;70:57–66. 33813026 10.1016/j.coi.2021.03.005 PMC8373719

[95] Liu XH, Zhai XY. Role of tryptophan metabolism in cancers and therapeutic implications. Biochimie. 2021;182:131–139. 33460767 10.1016/j.biochi.2021.01.005

[96] Solvay M, Holfelder P, Klaessens S, Pilotte L, Stroobant V, Lamy J, Naulaerts S, Spillier Q, Frédérick R, de Plaen E, et al. Tryptophan depletion sensitizes the AHR pathway by increasing AHR expression and GCN2/LAT1-mediated kynurenine uptake, and potentiates induction of regulatory T lymphocytes. J Immunother Cancer. 2023;11(6). 10.1136/jitc-2023-006728 PMC10314700 37344101

[97] Xue C, Li G, Zheng Q, Gu X, Shi Q, Su Y, Chu Q, Yuan X, Bao Z, Lu J, et al. Tryptophan metabolism in health and disease. Cell Metab. 2023;35(8):1304–1326. 37352864 10.1016/j.cmet.2023.06.004

[98] Du L, Xing Z, Tao B, Li T, Yang D, Li W, Zheng Y, Kuang C, Yan Q. Both IDO1 and TDO contribute to the malignancy of gliomas via the Kyn-AhR-AQP4 signaling pathway. Signal Transduct Target Ther. 2020;5(1):10. 32296044 10.1038/s41392-019-0103-4 PMC7033114

[99] Cheong JE, Sun L. Targeting the IDO1/TDO2–KYN–AhR pathway for cancer immunotherapy—Challenges and opportunities. Trends Pharmacol Sci. 2018;39(3):307–325. 29254698 10.1016/j.tips.2017.11.007

[100] Obara-Michlewska M. The tryptophan metabolism, kynurenine pathway and oxidative stress—Implications for glioma pathobiology. Neurochem Int. 2022;158: Article 105363. 35667490 10.1016/j.neuint.2022.105363

[101] Silvin A, Qian J, Ginhoux F. Brain macrophage development, diversity and dysregulation in health and disease. Cell Mol Immunol. 2023;20(11):1277–1289. 37365324 10.1038/s41423-023-01053-6 PMC10616292

[102] Ye Z, Ai X, Yang K, Yang Z, Fei F, Liao X, Qiu Z, Gimple RC, Yuan H, Huang H, et al. Targeting microglial metabolic rewiring synergizes with immune-checkpoint blockade therapy for glioblastoma. Cancer Discov. 2023;13(4):974–1001. 36649564 10.1158/2159-8290.CD-22-0455 PMC10073346

[103] Borst K, Dumas AA, Prinz M. Microglia: Immune and non-immune functions. Immunity. 2021;54(10):2194–2208. 34644556 10.1016/j.immuni.2021.09.014

[104] Lin H, Liu C, Hu A, Zhang D, Yang H, Mao Y. Understanding the immunosuppressive microenvironment of glioma: Mechanistic insights and clinical perspectives. J Hematol Oncol. 2024;17(1):31. 38720342 10.1186/s13045-024-01544-7 PMC11077829

[105] Veglia F, Sanseviero E, Gabrilovich DI. Myeloid-derived suppressor cells in the era of increasing myeloid cell diversity. Nat Rev Immunol. 2021;21(8):485–498. 33526920 10.1038/s41577-020-00490-y PMC7849958

[106] Wu Y, Yi M, Niu M, Mei Q, Wu K. Myeloid-derived suppressor cells: An emerging target for anticancer immunotherapy. Mol Cancer. 2022;21(1):184. 36163047 10.1186/s12943-022-01657-y PMC9513992

[107] Gao W, Wang X, Zhou Y, Wang X, Yu Y. Autophagy, ferroptosis, pyroptosis, and necroptosis in tumor immunotherapy. Signal Transduct Target Ther. 2022;7(1):196. 35725836 10.1038/s41392-022-01046-3 PMC9208265

[108] Wan S, Zhang G, Liu R, Abbas MN, Cui H. Pyroptosis, ferroptosis, and autophagy cross-talk in glioblastoma opens up new avenues for glioblastoma treatment. Cell Commun Signal. 2023;21(1):115. 37208730 10.1186/s12964-023-01108-1 PMC10199557

[109] Zhang X, Tang B, Luo J, Yang Y, Weng Q, Fang S, Zhao Z, Tu J, Chen M, Ji J. Cuproptosis, ferroptosis and PANoptosis in tumor immune microenvironment remodeling and immunotherapy: Culprits or new hope. Mol Cancer. 2024;23(1):255. 39543600 10.1186/s12943-024-02130-8 PMC11566504

[110] Liu R, Wang J, Liu Y, Gao Y, Yang R. Regulation of gut microbiota on immune cell ferroptosis: A novel insight for immunotherapy against tumor. Cancer Lett. 2024;598: Article 217115. 39025428 10.1016/j.canlet.2024.217115

[111] Chen J, Li T, Zhou N, He Y, Zhong J, Ma C, Zeng M, Ji J, Huang JD, Ke Y, et al. Engineered Salmonella inhibits GPX4 expression and induces ferroptosis to suppress glioma growth in vitro and in vivo. J Neuro-Oncol. 2023;163(3):607–622. 10.1007/s11060-023-04369-5 37351767

[112] Pan C, Ji Z, Wang Q, Zhang Z, Wang Z, Li C, Lu S, Ge P. Cuproptosis: Mechanisms, biological significance, and advances in disease treatment—A systematic review. CNS Neurosci Ther. 2024;30(9): Article e70039. 39267265 10.1111/cns.70039 PMC11392831

[113] Feng S, Zhang Y, Zhu H, Jian Z, Zeng Z, Ye Y, Li Y, Smerin D, Zhang X, Zou N, et al. Cuproptosis facilitates immune activation but promotes immune escape, and a machine learning-based cuproptosis-related signature is identified for predicting prognosis and immunotherapy response of gliomas. CNS Neurosci Ther. 2024;30(2): Article e14380. 37515314 10.1111/cns.14380 PMC10848101

[114] Zhang L, Huang S, Yuan Y. Butyrate inhibits the malignant biological behaviors of breast cancer cells by facilitating cuproptosis-associated gene expression. J Cancer Res Clin Oncol. 2024;150(6):287. 38833016 10.1007/s00432-024-05807-1 PMC11150186

[115] Bertheloot D, Latz E, Franklin BS. Necroptosis, pyroptosis and apoptosis: An intricate game of cell death. Cell Mol Immunol. 2021;18(5):1106–1121. 33785842 10.1038/s41423-020-00630-3 PMC8008022

[116] Yuan J, Ofengeim D. A guide to cell death pathways. Nat Rev Mol Cell Biol. 2024;25(5):379–395. 38110635 10.1038/s41580-023-00689-6

[117] Badgeley A, Anwar H, Modi K, Murphy P, Lakshmikuttyamma A. Effect of probiotics and gut microbiota on anti-cancer drugs: Mechanistic perspectives. Biochim Biophys Acta Rev Cancer. 2021;1875(1): Article 188494. 33346129 10.1016/j.bbcan.2020.188494

[118] Yao Z, Zhang X, Zhao F, Wang S, Chen A, Huang B, Wang J, Li X. Ursodeoxycholic acid inhibits glioblastoma progression via endoplasmic reticulum stress related apoptosis and synergizes with the proteasome inhibitor bortezomib. ACS Chem Neurosci. 2020;11(9):1337–1346. 32239921 10.1021/acschemneuro.0c00095

[119] Wang Y, du J, Wu X, Abdelrehem A, Ren Y, Liu C, Zhou X, Wang S. Crosstalk between autophagy and microbiota in cancer progression. Mol Cancer. 2021;20(1):163. 34895252 10.1186/s12943-021-01461-0 PMC8665582

[120] Feng F, Zhang M, Yang C, Heng X, Wu X. The dual roles of autophagy in gliomagenesis and clinical therapy strategies based on autophagic regulation mechanisms. Biomed Pharmacother. 2019;120: Article 109441. 31541887 10.1016/j.biopha.2019.109441

[121] Li X, He S, Ma B. Autophagy and autophagy-related proteins in cancer. Mol Cancer. 2020;19(1):12. 31969156 10.1186/s12943-020-1138-4 PMC6975070

[122] Goubet AG, Wheeler R, Fluckiger A, Qu B, Lemaître F, Iribarren K, Mondragón L, Tidjani Alou M, Pizzato E, Durand S, et al. Multifaceted modes of action of the anticancer probiotic Enterococcus hirae. Cell Death Differ. 2021;28(7):2276–2295. 33976389 10.1038/s41418-021-00753-8 PMC8257780

[123] Ren Z, Chen S, Lv H, Peng L, Yang W, Chen J, Wu Z, Wan C. Effect of Bifidobacterium animalis subsp. lactis SF on enhancing the tumor suppression of irinotecan by regulating the intestinal flora. Pharmacol Res. 2022;184: Article 106406. 35987480 10.1016/j.phrs.2022.106406

[124] Feng S, Wan Q, Wu W, Zhang C, Lu H, Lu X. Effect of gut microbiome regulated Taohong Siwu Decoction metabolism on glioma cell phenotype. Front Cell Infect Microbiol. 2023;13:1192589. 37342242 10.3389/fcimb.2023.1192589 PMC10277651

[125] Kennedy LB, Salama AKS. A review of cancer immunotherapy toxicity. CA Cancer J Clin. 2020;70(2):86–104. 31944278 10.3322/caac.21596

[126] Barbari C, Fontaine T, Parajuli P, Lamichhane N, Jakubski S, Lamichhane P, Deshmukh RR. Immunotherapies and combination strategies for immuno-oncology. Int J Mol Sci. 2020;21(14):5009. 32679922 10.3390/ijms21145009 PMC7404041

[127] Zhang Y, Zhang Z. The history and advances in cancer immunotherapy: Understanding the characteristics of tumor-infiltrating immune cells and their therapeutic implications. Cell Mol Immunol. 2020;17(8):807–821. 32612154 10.1038/s41423-020-0488-6 PMC7395159

[128] Lei X, Lei Y, Li JK, du WX, Li RG, Yang J, Li J, Li F, Tan HB. Immune cells within the tumor microenvironment: Biological functions and roles in cancer immunotherapy. Cancer Lett. 2020;470:126–133. 31730903 10.1016/j.canlet.2019.11.009

[129] Salinas RD, Durgin JS, O’Rourke DM. Potential of glioblastoma-targeted chimeric antigen receptor (CAR) T-cell therapy. CNS Drugs. 2020;34(2):127–145. 31916100 10.1007/s40263-019-00687-3

[130] Lin YJ, Mashouf LA, Lim M. CAR T cell therapy in primary brain tumors: Current investigations and the future. Front Immunol. 2022;13: Article 817296. 35265074 10.3389/fimmu.2022.817296 PMC8899093

[131] Donovan LK, Delaidelli A, Joseph SK, Bielamowicz K, Fousek K, Holgado BL, Manno A, Srikanthan D, Gad AZ, van Ommeren R, et al. Locoregional delivery of CAR T cells to the cerebrospinal fluid for treatment of metastatic medulloblastoma and ependymoma. Nat Med. 2020;26(5):720–731. 32341580 10.1038/s41591-020-0827-2 PMC8815773

[132] Upreti D, Bakhshinyan D, Bloemberg D, Vora P, Venugopal C, Singh SK. Strategies to enhance the efficacy of T-cell therapy for central nervous system tumors. Front Immunol. 2020;11: Article 599253. 33281826 10.3389/fimmu.2020.599253 PMC7689359

[133] Chang Y, Cai X, Syahirah R, Yao Y, Xu Y, Jin G, Bhute VJ, Torregrosa-Allen S, Elzey BD, Won YY, et al. CAR-neutrophil mediated delivery of tumor-microenvironment responsive nanodrugs for glioblastoma chemo-immunotherapy. Nat Commun. 2023;14(1):2266. 37080958 10.1038/s41467-023-37872-4 PMC10119091

[134] Burger MC, Zhang C, Harter PN, Romanski A, Strassheimer F, Senft C, Tonn T, Steinbach JP, Wels WS. CAR-engineered NK cells for the treatment of glioblastoma: Turning innate effectors into precision tools for cancer immunotherapy. Front Immunol. 2019;10:2683. 31798595 10.3389/fimmu.2019.02683 PMC6868035

[135] Gao L, Shi C, Yang Z, Jing W, Han M, Zhang J, Zhang C, Tang C, Dong Y, Liu Y, et al. Convection-enhanced delivery of nanoencapsulated gene locoregionally yielding ErbB2/Her2-specific CAR-macrophages for brainstem glioma immunotherapy. J Nanobiotechnol. 2023;21(1):56. 10.1186/s12951-023-01810-9 PMC9940362 36805678

[136] Ghouzlani A, Kandoussi S, Tall M, Reddy KP, Rafii S, Badou A. Immune checkpoint inhibitors in human glioma microenvironment. Front Immunol. 2021;12: Article 679425. 34305910 10.3389/fimmu.2021.679425 PMC8301219

[137] Yasinjan F, Xing Y, Geng H, Guo R, Yang L, Liu Z, Wang H. Immunotherapy: A promising approach for glioma treatment. Front Immunol. 2023;14:1255611. 37744349 10.3389/fimmu.2023.1255611 PMC10512462

[138] Jiacheng D, Jiayue C, Ying G, Shaohua W, Wenhui L, Xinyu H. Research progress and challenges of the PD-1/PD-L1 axis in gliomas. Cell Biosci. 2024;14(1):123. 39334448 10.1186/s13578-024-01305-6 PMC11437992

[139] Cai L, Li Y, Tan J, Xu L, Li Y. Targeting LAG-3, TIM-3, and TIGIT for cancer immunotherapy. J Hematol Oncol. 2023;16(1):101. 37670328 10.1186/s13045-023-01499-1 PMC10478462

[140] Fu J, Mao L, Jiao Y, Mei D, Chen Y. Elucidating CTLA-4’s role in tumor immunity: A comprehensive overview of targeted antibody therapies and clinical developments. Mol Divers. 2024. 10.1007/s11030-024-10917-6 38985379

[141] Hossen MM, Ma Y, Yin Z, Xia Y, du J, Huang JY, Huang JJ, Zou L, Ye Z, Huang Z. Current understanding of CTLA-4: From mechanism to autoimmune diseases. Front Immunol. 2023;14:1198365. 37497212 10.3389/fimmu.2023.1198365 PMC10367421

[142] Raphael I, Kumar R, McCarl LH, Shoger K, Wang L, Sandlesh P, Sneiderman CT, Allen J, Zhai S, Campagna ML, et al. TIGIT and PD-1 immune checkpoint pathways are associated with patient outcome and anti-tumor immunity in glioblastoma. Front Immunol. 2021;12: Article 637146. 34025646 10.3389/fimmu.2021.637146 PMC8137816

[143] Anderson AC, Joller N, Kuchroo VK. Lag-3, Tim-3, and TIGIT: Co-inhibitory receptors with specialized functions in immune regulation. Immunity. 2016;44(5):989–1004. 27192565 10.1016/j.immuni.2016.05.001 PMC4942846

[144] Hu W, Li D, Yang Y, Zheng Y, Zeng J, Sai K. TIM-3/CD68 double-high expression in glioma: Prognostic characteristics and potential therapeutic approaches. Int Immunopharmacol. 2024;139: Article 112665. 39002523 10.1016/j.intimp.2024.112665

[145] Dixon KO, Lahore GF, Kuchroo VK. Beyond T cell exhaustion: TIM-3 regulation of myeloid cells. Sci Immunol. 2024;9(93): Article eadf2223. 38457514 10.1126/sciimmunol.adf2223

[146] Zhang P, Liu X, Gu Z, Jiang Z, Zhao S, Song Y, Yu J. Targeting TIGIT for cancer immunotherapy: Recent advances and future directions. Biomark Res. 2024;12(1):7. 38229100 10.1186/s40364-023-00543-z PMC10790541

[147] Chu X, Tian W, Wang Z, Zhang J, Zhou R. Co-inhibition of TIGIT and PD-1/PD-L1 in cancer immunotherapy: Mechanisms and clinical trials. Mol Cancer. 2023;22(1):93. 37291608 10.1186/s12943-023-01800-3 PMC10249258

[148] Matlung HL, Szilagyi K, Barclay NA, van den Berg TK. The CD47-SIRPα signaling axis as an innate immune checkpoint in cancer. Immunol Rev. 2017;276(1):145–164. 28258703 10.1111/imr.12527

[149] Bach N, Winzer R, Tolosa E, Fiedler W, Brauneck F. The clinical significance of CD73 in cancer. Int J Mol Sci. 2023;24(14):11759. 37511518 10.3390/ijms241411759 PMC10380759

[150] Lee AH, Sun L, Mochizuki AY, Reynoso JG, Orpilla J, Chow F, Kienzler JC, Everson RG, Nathanson DA, Bensinger SJ, et al. Neoadjuvant PD-1 blockade induces T cell and cDC1 activation but fails to overcome the immunosuppressive tumor associated macrophages in recurrent glioblastoma. Nat Commun. 2021;12(1):6938. 34836966 10.1038/s41467-021-26940-2 PMC8626557

[151] Zeng YF, Wei XY, Guo QH, Chen SY, Deng S, Liu ZZ, Gong ZC, Zeng WJ. The efficacy and safety of anti-PD-1/PD-L1 in treatment of glioma: A single-arm meta-analysis. Front Immunol. 2023;14:1168244. 37122727 10.3389/fimmu.2023.1168244 PMC10140424

[152] Guo D, Tong Y, Jiang X, Meng Y, Jiang H, du L, Wu Q, Li S, Luo S, Li M, et al. Aerobic glycolysis promotes tumor immune evasion by hexokinase2-mediated phosphorylation of IκBα. Cell Metab. 2022;34(9):1312–1324.e6. 36007522 10.1016/j.cmet.2022.08.002

[153] Dmello C, Zhao J, Chen L, Gould A, Castro B, Arrieta VA, Zhang DY, Kim KS, Kanojia D, Zhang P, et al. Checkpoint kinase 1/2 inhibition potentiates anti-tumoral immune response and sensitizes gliomas to immune checkpoint blockade. Nat Commun. 2023;14(1):1566. 36949040 10.1038/s41467-023-36878-2 PMC10033639

[154] Mei Y, Wang X, Zhang J, Liu D, He J, Huang C, Liao J, Wang Y, Feng Y, Li H, et al. Siglec-9 acts as an immune-checkpoint molecule on macrophages in glioblastoma, restricting T-cell priming and immunotherapy response. Nat Cancer. 2023;4(9):1273–1291. 37460871 10.1038/s43018-023-00598-9

[155] Huang H, Georganaki M, Conze LL, Laviña B, van Hooren L, Vemuri K, van de Walle T, Ramachandran M, Zhang L, Pontén F, et al. ELTD1 deletion reduces vascular abnormality and improves T-cell recruitment after PD-1 blockade in glioma. Neuro-Oncology. 2022;24(3):398–411. 34347079 10.1093/neuonc/noab181 PMC8917395

[156] Liu H, Zhao Q, Tan L, Wu X, Huang R, Zuo Y, Chen L, Yang J, Zhang ZX, Ruan W, et al. Neutralizing IL-8 potentiates immune checkpoint blockade efficacy for glioma. Cancer Cell. 2023;41(4):693–710.e8. 36963400 10.1016/j.ccell.2023.03.004

[157] Li R, Zhan Y, Ding X, Cui J, Han Y, Zhang J, Zhang J, Li W, Wang L, Jiang J. Cancer differentiation inducer chlorogenic acid suppresses PD-L1 expression and boosts antitumor immunity of PD-1 antibody. Int J Biol Sci. 2024;20(1):61–77. 38164171 10.7150/ijbs.83599 PMC10750284

[158] Li T, Xu D, Ruan Z, Zhou J, Sun W, Rao B, Xu H. Metabolism/immunity dual-regulation thermogels potentiating immunotherapy of glioblastoma through lactate-excretion inhibition and PD-1/PD-L1 blockade. Adv Sci. 2024;11(18): Article e2310163. 10.1002/advs.202310163 PMC11095231 38460167

[159] Kim J, Kim Y, la J, Park WH, Kim HJ, Park SH, Ku KB, Kang BH, Lim J, Kwon MS, et al. Supplementation with a high-glucose drink stimulates anti-tumor immune responses to glioblastoma via gut microbiota modulation. Cell Rep. 2023;42(10): Article 113220. 37804509 10.1016/j.celrep.2023.113220

[160] Tritz ZP, Ayasoufi K, Wolf DM, Owens CA, Malo CS, Himes BT, Fain CE, Goddery EN, Yokanovich LT, Jin F, et al. Anti-PD-1 and extended half-life IL2 synergize for treatment of murine glioblastoma independent of host MHC class I expression. Cancer Immunol Res. 2023;11(6):763–776. 36921098 10.1158/2326-6066.CIR-22-0570 PMC10239322

[161] Nassiri F, Patil V, Yefet LS, Singh O, Liu J, Dang RMA, Yamaguchi TN, Daras M, Cloughesy TF, Colman H, et al. Oncolytic DNX-2401 virotherapy plus pembrolizumab in recurrent glioblastoma: A phase 1/2 trial. Nat Med. 2023;29(6):1370–1378. 37188783 10.1038/s41591-023-02347-y PMC10287560

[162] Arrieta VA, Gould A, Kim KS, Habashy KJ, Dmello C, Vázquez-Cervantes GI, Palacín-Aliana I, McManus G, Amidei C, Gomez C, et al. Ultrasound-mediated delivery of doxorubicin to the brain results in immune modulation and improved responses to PD-1 blockade in gliomas. Nat Commun. 2024;15(1):4698. 38844770 10.1038/s41467-024-48326-w PMC11156895

[163] Chen Q, Zheng Y, Chen X, Xing Y, Zhang J, Yan X, Zhang Q, Wu D, Chen Z. Bacteria synergized with PD-1 blockade enhance positive feedback loop of cancer cells-M1 macrophages-T cells in glioma. Adv Sci. 2024;11(20): Article e2308124. 10.1002/advs.202308124 PMC11132069 38520726

[164] Yang Y, Luo X, Wang Y, Xu A, Peng L, Zhang X, Wang Z, Ying Y, Li K. β-Mangostin targets and suppresses glioma via STING activation and tumor-associated microglia polarization. Biomed Pharmacother. 2024;177: Article 117074. 38972149 10.1016/j.biopha.2024.117074

[165] Park J, Park SA, Kim YS, Kim D, Shin S, Lee SH, Jeun SS, Chung YJ, Ahn S. Intratumoral IL-12 delivery via mesenchymal stem cells combined with PD-1 blockade leads to long-term antitumor immunity in a mouse glioblastoma model. Biomed Pharmacother. 2024;173: Article 115790. 38431436 10.1016/j.biopha.2023.115790

[166] Datta M, Chatterjee S, Perez EM, Gritsch S, Roberge S, Duquette M, Chen IX, Naxerova K, Kumar AS, Ghosh M, et al. Losartan controls immune checkpoint blocker-induced edema and improves survival in glioblastoma mouse models. Proc Natl Acad Sci USA. 2023;120(6): Article e2219199120. 36724255 10.1073/pnas.2219199120 PMC9963691

[167] Chen D, Varanasi SK, Hara T, Traina K, Sun M, McDonald B, Farsakoglu Y, Clanton J, Xu S, Garcia-Rivera L, et al. CTLA-4 blockade induces a microglia-Th1 cell partnership that stimulates microglia phagocytosis and anti-tumor function in glioblastoma. Immunity. 2023;56(9):2086–2104.e8. 37572655 10.1016/j.immuni.2023.07.015 PMC11800830

[168] Kim KS, Habashy K, Gould A, Zhao J, Najem H, Amidei C, Saganty R, Arrieta VA, Dmello C, Chen L, et al. Fc-enhanced anti-CTLA-4, anti-PD-1, doxorubicin, and ultrasound-mediated blood-brain barrier opening: A novel combinatorial immunotherapy regimen for gliomas. Neuro-Oncology. 2024;26(11):2044–2060. 39028616 10.1093/neuonc/noae135 PMC11534315

[169] Lee J, Lathia JD. The one-two punch: TIM-3 blockade targets immune and tumor cells to knock out pediatric brain tumors. Cancer Cell. 2023;41(11):1843–1845. 37863067 10.1016/j.ccell.2023.09.016

[170] Lupo KB, Yao X, Borde S, Wang J, Torregrosa-Allen S, Elzey BD, Utturkar S, Lanman NA, McIntosh MK, Matosevic S. synNotch-programmed iPSC-derived NK cells usurp TIGIT and CD73 activities for glioblastoma therapy. Nat Commun. 2024;15(1):1909. 38429294 10.1038/s41467-024-46343-3 PMC10907695

[171] Ye L, Lv W, He W, Li S, Min Z, Gong L, Zhang Q, Teng C, Sun S, Lv L, et al. Reduced malignant glioblastoma recurrence post-resection through the anti-CD47 antibody and Temozolomide co-embedded in-situ hydrogel system. J Control Release. 2023;359:224–233. 37290721 10.1016/j.jconrel.2023.05.046

[172] Du L, Su Z, Wang S, Meng Y, Xiao F, Xu D, Li X, Qian X, Lee SB, Lee J-H, et al. EGFR-induced and c-Src-mediated CD47 phosphorylation inhibits TRIM21-dependent polyubiquitylation and degradation of CD47 to promote tumor immune evasion. Adv Sci. 2023;10(27): Article e2206380. 10.1002/advs.202206380 PMC10520678 37541303

[173] Sun T, Liu B, Cao Y, Li Y, Cai L, Yang. AMPK-mediated CD47 H3K4 methylation promotes phagocytosis evasion of glioma stem cells post-radiotherapy. Cancer Lett. 2024;583: Article 216605. 38218171 10.1016/j.canlet.2023.216605

[174] Marquardt V, Theruvath J, Pauck D, Picard D, Qin N, Blümel L, Maue M, Bartl J, Ahmadov U, Langini M, et al. Tacedinaline (CI-994), a class I HDAC inhibitor, targets intrinsic tumor growth and leptomeningeal dissemination in MYC-driven medulloblastoma while making them susceptible to anti-CD47-induced macrophage phagocytosis via NF-kB-TGM2 driven tumor inflammation. J Immunother Cancer. 2023;11(1): Article e005871. 36639156 10.1136/jitc-2022-005871 PMC9843227

[175] Sheybani ND, Breza VR, Paul S, McCauley KS, Berr SS, Miller GW, Neumann KD, Price RJ. ImmunoPET-informed sequence for focused ultrasound-targeted mCD47 blockade controls glioma. J Control Release. 2021;331:19–29. 33476735 10.1016/j.jconrel.2021.01.023 PMC7946780

[176] Liu X, Zhang H, Wang C, Li Z, Zhu Q, Feng Y, Fan J, Qi S, Wu Z, Liu Y. Tumor-selective blockade of CD47 signaling with CD47 antibody for enhanced anti-tumor activity in malignant meningioma. Curr Neuropharmacol. 2023;21(10):2159–2173. 37171006 10.2174/1570159X21666230511123157 PMC10556363

[177] Zhang H, Yang L, Han M, Han Y, Jiang Z, Zheng Q, Dong J, Wang T, Li Z. Boost infiltration and activity of T cells via inhibiting ecto-5’-nucleotidase (CD73) immune checkpoint to enhance glioblastoma immunotherapy. ACS Nano. 2024;18(34):23001–23013. 39150454 10.1021/acsnano.4c04553

[178] Azambuja JH, Schuh RS, Michels LR, Gelsleichter NE, Beckenkamp LR, Iser IC, Lenz GS, de Oliveira FH, Venturin G, Greggio S, et al. Nasal administration of cationic nanoemulsions as CD73-siRNA delivery system for glioblastoma treatment: A new therapeutical approach. Mol Neurobiol. 2020;57(2):635–649. 31407144 10.1007/s12035-019-01730-6

[179] Efimova I, Catanzaro E, van der Meeren L, Turubanova VD, Hammad H, Mishchenko TA, Vedunova MV, Fimognari C, Bachert C, Coppieters F, et al. Vaccination with early ferroptotic cancer cells induces efficient antitumor immunity. J Immunother Cancer. 2020;8(2): Article e001369. 33188036 10.1136/jitc-2020-001369 PMC7668384

[180] Zhan S, Lu L, Pan SS, Wei XQ, Miao RR, Liu XH, Xue M, Lin XK, Xu HL. Targeting NQO1/GPX4-mediated ferroptosis by plumbagin suppresses in vitro and in vivo glioma growth. Br J Cancer. 2022;127(2):364–376. 35396498 10.1038/s41416-022-01800-y PMC9296534

[181] Sun S, Qi G, Chen H, He D, Ma D, Bie Y, Xu L, Feng B, Pang Q, Guo H, et al. Ferroptosis sensitization in glioma: Exploring the regulatory mechanism of SOAT1 and its therapeutic implications. Cell Death Dis. 2023;14(11):754. 37980334 10.1038/s41419-023-06282-1 PMC10657441

[182] Upadhyayula PS, Higgins DM, Mela A, Banu M, Dovas A, Zandkarimi F, Patel P, Mahajan A, Humala N, Nguyen TTT, et al. Dietary restriction of cysteine and methionine sensitizes gliomas to ferroptosis and induces alterations in energetic metabolism. Nat Commun. 2023;14(1):1187. 36864031 10.1038/s41467-023-36630-w PMC9981683

[183] Li K, Chen B, Xu A, Shen J, Li K, Hao K, Hao R, Yang W, Jiang W, Zheng Y, et al. TRIM7 modulates NCOA4-mediated ferritinophagy and ferroptosis in glioblastoma cells. Redox Biol. 2022;56: Article 102451. 36067704 10.1016/j.redox.2022.102451 PMC9468590

[184] Miao Z, Xu L, Gu W, Ren Y, Li R, Zhang S, Chen C, Wang H, Ji J, Chen J. A targetable PRR11-DHODH axis drives ferroptosis- and temozolomide-resistance in glioblastoma. Redox Biol. 2024;73: Article 103220. 38838551 10.1016/j.redox.2024.103220 PMC11179629

[185] Miao Z, Tian W, Ye Y, Gu W, Bao Z, Xu L, Sun G, Li C, Tu Y, Chao H, et al. Hsp90 induces Acsl4-dependent glioma ferroptosis via dephosphorylating Ser637 at Drp1. Cell Death Dis. 2022;13(6):548. 35697672 10.1038/s41419-022-04997-1 PMC9192632

[186] Li X, Zhang W, Xing Z, Hu S, Zhang G, Wang T, Wang T, Fan Q, Chen G, Cheng J, et al. Targeting SIRT3 sensitizes glioblastoma to ferroptosis by promoting mitophagy and inhibiting SLC7A11. Cell Death Dis. 2024;15(2):168. 38395990 10.1038/s41419-024-06558-0 PMC10891132

[187] Li X, Cheng Y, Yang Z, Ji Q, Huan M, Ye W, Liu M, Zhang B, Liu D, Zhou S. Glioma-targeted oxaliplatin/ferritin clathrate reversing the immunosuppressive microenvironment through hijacking Fe^2+^ and boosting Fenton reaction. J Nanobiotechnol. 2024;22(1):93. 10.1186/s12951-024-02376-w PMC10913265 38443927

[188] Zhang S, Yu H, Sun S, Fan X, Bi W, Li S, Wang W, Fang Z, Chen X. Copper homeostasis based on cuproptosis-related signature optimizes molecular subtyping and treatment of glioma. Mol Neurobiol. 2024;61(8):4962–4975. 38151613 10.1007/s12035-023-03893-9

[189] Zhu Y, Niu X, Ding C, Lin Y, Fang W, Yan L, Cheng J, Zou J, Tian Y, Huang W, et al. Carrier-free self-assembly nano-sonosensitizers for sonodynamic-amplified cuproptosis-ferroptosis in glioblastoma therapy. Adv Sci. 2024;11(23): Article e2402516. 10.1002/advs.202402516 PMC11187904 38582500

[190] Wu L, Lin H, Cao X, Tong Q, Yang F, Miao Y, Ye D, Fan Q. Bioorthogonal Cu single-atom nanozyme for synergistic nanocatalytic therapy, photothermal therapy, cuproptosis and immunotherapy. Angew Chem Int Ed Engl. 2024;63(27): Article e202405937. 38654446 10.1002/anie.202405937

[191] Dai L, Zhou P, Lyu L, Jiang S. Systematic analysis based on the cuproptosis-related genes identifies ferredoxin 1 as an immune regulator and therapeutic target for glioblastoma. BMC Cancer. 2023;23(1):1249. 38114959 10.1186/s12885-023-11727-z PMC10731758

[192] Cheng M, Liu Y, You Q, Lei Z, Ji J, Zhang F, Dong WF, Li L. Metal-doping strategy for carbon-based sonosensitizer in sonodynamic therapy of glioblastoma. Adv Sci. 2024;11(34): Article e2404230. 10.1002/advs.202404230 PMC11425966 38984451

[193] Pandey S, Lee M, Lim J, Park S, Choung YH, Kim JE, Garg P, Chung JH. SMO-CRISPR-mediated apoptosis in CD133-targeted cancer stem cells and tumor growth inhibition. J Control Release. 2023;357:94–108. 36931470 10.1016/j.jconrel.2023.03.023

[194] Chen J, Liu Z, Fang H, Su Q, Fan Y, Song L, He S. Therapeutic efficacy of a novel self-assembled immunostimulatory siRNA combining apoptosis promotion with RIG-I activation in gliomas. J Transl Med. 2024;22(1):395. 38685028 10.1186/s12967-024-05151-5 PMC11057130

[195] Wang W, Yuan X, Mu J, Zou Y, Xu L, Chen J, Zhu X, Li B, Zeng Z, Wu X, et al. Quercetin induces MGMT^+^ glioblastoma cells apoptosis via dual inhibition of Wnt3a/β-catenin and Akt/NF-κB signaling pathways. Phytomedicine. 2023;118: Article 154933. 37451151 10.1016/j.phymed.2023.154933

[196] Damhofer H, Tatar T, Southgate B, Scarneo S, Agger K, Shlyueva D, Uhrbom L, Morrison GM, Hughes PF, Haystead T, et al. TAK1 inhibition leads to RIPK1-dependent apoptosis in immune-activated cancers. Cell Death Dis. 2024;15(4):273. 38632238 10.1038/s41419-024-06654-1 PMC11024179

[197] He W, Li X, Morsch M, Ismail M, Liu Y, Rehman FU, Zhang D, Wang Y, Zheng M, Chung R, et al. Brain-targeted codelivery of Bcl-2/Bcl-xl and Mcl-1 inhibitors by biomimetic nanoparticles for orthotopic glioblastoma therapy. ACS Nano. 2022;16(4):6293–6308. 35353498 10.1021/acsnano.2c00320

[198] Guo T, Wu C, Zhou L, Zhang J, Wang W, Shen Y, Zhang L, Niu M, Zhang X, Yu R, et al. Preclinical evaluation of Mito-LND, a targeting mitochondrial metabolism inhibitor, for glioblastoma treatment. J Transl Med. 2023;21(1):532. 37550679 10.1186/s12967-023-04332-y PMC10405494

[199] Madhavan K, Balakrishnan I, Lakshmanachetty S, Pierce A, Sanford B, Fosmire S, Elajaili HB, Walker F, Wang D, Nozik ES, et al. Venetoclax cooperates with ionizing radiation to attenuate diffuse midline glioma tumor growth. Clin Cancer Res. 2022;28(11):2409–2424. 35344040 10.1158/1078-0432.CCR-21-4002

[200] Li Z, Fu WJ, Chen XQ, Wang S, Deng RS, Tang XP, Yang KD, Niu Q, Zhou H, Li QR, et al. Autophagy-based unconventional secretion of HMGB1 in glioblastoma promotes chemosensitivity to temozolomide through macrophage M1-like polarization. J Exp Clin Cancer Res. 2022;41(1):74. 35193644 10.1186/s13046-022-02291-8 PMC8862393

[201] Chryplewicz A, Scotton J, Tichet M, Zomer A, Shchors K, Joyce JA, Homicsko K, Hanahan D. Cancer cell autophagy, reprogrammed macrophages, and remodeled vasculature in glioblastoma triggers tumor immunity. Cancer Cell. 2022;40(10):1111–1127.e9. 36113478 10.1016/j.ccell.2022.08.014 PMC9580613

[202] Jia W, Tian H, Jiang J, Zhou L, Li L, Luo M, Ding N, Nice EC, Huang C, Zhang H. Brain-targeted HFn-Cu-REGO nanoplatform for site-specific delivery and manipulation of autophagy and cuproptosis in glioblastoma. Small. 2023;19(2): Article e2205354. 36399643 10.1002/smll.202205354

[203] Xing Y, Wei X, Liu Y, Wang MM, Sui Z, Wang X, Zhu W, Wu M, Lu C, Fei YH, et al. Autophagy inhibition mediated by MCOLN1/TRPML1 suppresses cancer metastasis via regulating a ROS-driven TP53/p53 pathway. Autophagy. 2022;18(8):1932–1954. 34878954 10.1080/15548627.2021.2008752 PMC9450983

[204] Ou M, Cho HY, Fu J, Thein TZ, Wang W, Swenson SD, Minea RO, Stathopoulos A, Schönthal AH, Hofman FM, et al. Inhibition of autophagy and induction of glioblastoma cell death by NEO214, a perillyl alcohol-rolipram conjugate. Autophagy. 2023;19(12):3169–3188. 37545052 10.1080/15548627.2023.2242696 PMC10621246

[205] Meng Y, Sun J, Zhang G, Yu T, Piao H. Bacteria associated with glioma: A next wave in cancer treatment. Front Cell Infect Microbiol. 2023;13:1164654. 37201117 10.3389/fcimb.2023.1164654 PMC10185885

[206] Gurbatri CR, Lia I, Vincent R, Coker C, Castro S, Treuting PM, Hinchliffe TE, Arpaia N, Danino T. Engineered probiotics for local tumor delivery of checkpoint blockade nanobodies. Sci Transl Med. 2020;12(530): Article eaax0876. 32051224 10.1126/scitranslmed.aax0876 PMC7685004

[207] Zhang Y, Xi K, Fu Z, Zhang Y, Cheng B, Feng F, Dong Y, Fang Z, Zhang Y, Shen J, et al. Stimulation of tumoricidal immunity via bacteriotherapy inhibits glioblastoma relapse. Nat Commun. 2024;15(1):4241. 38762500 10.1038/s41467-024-48606-5 PMC11102507

[208] Chen Z, Yong T, Wei Z, Zhang X, Li X, Qin J, Li J, Hu J, Yang X, Gan L. Engineered probiotic-based personalized cancer vaccine potentiates antitumor immunity through initiating trained immunity. Adv Sci. 2024;11(3): Article e2305081. 10.1002/advs.202305081 PMC10797439 38009498

[209] Miao YB, Zhao W, Renchi G, Gong Y, Shi Y. Customizing delivery nano-vehicles for precise brain tumor therapy. J Nanobiotechnol. 2023;21(1):32. 10.1186/s12951-023-01775-9 PMC9883977 36707835

[210] Yin Y, Wang J, Yang M, du R, Pontrelli G, McGinty S, Wang G, Yin T, Wang Y. Penetration of the blood-brain barrier and the anti-tumour effect of a novel PLGA-lysoGM1/DOX micelle drug delivery system. Nanoscale. 2020;12(5):2946–2960. 31994576 10.1039/c9nr08741a

[211] Gu J, Liu X, Ji Z, Shen M, Zhu M, Ren Y, Guo L, Yang K, Liu T, Yi X. Tumor vascular destruction and cGAS-STING activation induced by single drug-loaded nano-micelles for multiple synergistic therapies of cancer. Small. 2023;19(46): Article e2303517. 37475514 10.1002/smll.202303517

[212] Wu S, Lu L, Zhou J, Ran D, Wang S, Xu Q, Xu W, Wang J, Liu Y, Xie C, et al. All-stage targeted therapy for glioblastoma based on lipid membrane coated cabazitaxel nanocrystals. J Control Release. 2022;345:685–695. 35346767 10.1016/j.jconrel.2022.03.047

[213] Wu T, Liu Y, Cao Y, Liu Z. Engineering macrophage exosome disguised biodegradable nanoplatform for enhanced sonodynamic therapy of glioblastoma. Adv Mater. 2022;34(15): Article e2110364. 35133042 10.1002/adma.202110364

[214] Zhang J, Han L, Wu H, Zhong Y, Shangguan P, Liu Y, He M, Sun H, Song C, Wang X, et al. A brain-targeting NIR-II ferroptosis system: Effective visualization and oncotherapy for orthotopic glioblastoma. Adv Sci. 2023;10(13): Article e2206333. 10.1002/advs.202206333 PMC10161027 36869410

[215] Chung S, Sugimoto Y, Huang J, Zhang M. Iron oxide nanoparticles decorated with functional peptides for a targeted siRNA delivery to glioma cells. ACS Appl Mater Interfaces. 2023;15(1):106–119. 36442077 10.1021/acsami.2c17802 PMC11495154

[216] Zhu N, Chen S, Jin Y, Wang M, Fang L, Xue L, Hua D, Zhang Z, Jia M, Hao M, et al. Enhancing glioblastoma immunotherapy with integrated chimeric antigen receptor T cells through the re-education of tumor-associated microglia and macrophages. ACS Nano. 2024;18(17):11165–11182. 38626338 10.1021/acsnano.4c00050

[217] Wang H, Chao Y, Zhao H, Zhou X, Zhang F, Zhang Z, Li Z, Pan J, Wang J, Chen Q, et al. Smart nanomedicine to enable crossing blood-brain barrier delivery of checkpoint blockade antibody for immunotherapy of glioma. ACS Nano. 2022;16(1):664–674. 34978418 10.1021/acsnano.1c08120

[218] Nie, W., Wu, G., Zhang, J., Huang, L., Ding, J., Jiang, A., Zhang, Y., Liu, Y., Li, J., Pu, K., Xie, H. Responsive Exosome Nano-bioconjugates for Synergistic Cancer Therapy. Angewandte Chemie International Edition. 2019; 59(5):2018-2022. Portico. https://doi.org/10.1002/anie.201912524 31746532 10.1002/anie.201912524

[219] Wang R, Song W, Zhu J, Shao X, Yang C, Xiong W, Wang B, Zhao P, Chen M, Huang Y. Biomimetic nano-chelate diethyldithiocarbamate Cu/Fe for enhanced metalloimmunity and ferroptosis activation in glioma therapy. J Control Release. 2024;368:84–96. 38331004 10.1016/j.jconrel.2024.02.004

[220] Xu Q, Zhang H, Liu H, Han Y, Qiu W, Li Z. Inhibiting autophagy flux and DNA repair of tumor cells to boost radiotherapy of orthotopic glioblastoma. Biomaterials. 2022;280: Article 121287. 34864449 10.1016/j.biomaterials.2021.121287

[221] Wu J, Heidelberg RE, Gajjar A. Adolescents and young adults with cancer: CNS tumors. J Clin Oncol. 2024;42(6):686–695. 38064656 10.1200/JCO.23.01747 PMC11550794

[222] Sampson JH, Gunn MD, Fecci PE, Ashley DM. Brain immunology and immunotherapy in brain tumours. Nat Rev Cancer. 2020;20(1):12–25. 31806885 10.1038/s41568-019-0224-7 PMC7327710

[223] BharathwajChetty B, Kumar A, Deevi P, Abbas M, Alqahtani A, Liang L, Sethi G, Liu L, Kunnumakkara AB. Gut microbiota and their influence in brain cancer milieu. J Neuroinflammation. 2025;22(1):129. 40312370 10.1186/s12974-025-03434-2 PMC12046817

[224] Yang J, Lu J, Dong Y, Wei Y, Christian M, Huang J, Kuang H, Cao D. Revealing the link between gut microbiota and brain tumor risk: A new perspective from Mendelian randomization. Front Cell Infect Microbiol. 2024;14:1404745. 39165915 10.3389/fcimb.2024.1404745 PMC11333460

[225] Wang JZ, Landry AP, Raleigh DR, Sahm F, Walsh KM, Goldbrunner R, Yefet LS, Tonn JC, Gui C, Ostrom QT, et al. Meningioma: International Consortium on Meningiomas consensus review on scientific advances and treatment paradigms for clinicians, researchers, and patients. Neuro-Oncology. 2024;26(10):1742–1780. 38695575 10.1093/neuonc/noae082 PMC11449035

